# Randomized trial of neoadjuvant vaccination with tumor-cell lysate induces T cell response in low-grade gliomas

**DOI:** 10.1172/JCI151239

**Published:** 2022-02-01

**Authors:** Hirokazu Ogino, Jennie W. Taylor, Takahide Nejo, David Gibson, Payal B. Watchmaker, Kaori Okada, Atsuro Saijo, Meghan R. Tedesco, Anny Shai, Cynthia M. Wong, Jane E. Rabbitt, Michael R. Olin, Christopher L. Moertel, Yasuhiko Nishioka, Andres M. Salazar, Annette M. Molinaro, Joanna J. Phillips, Nicholas A. Butowski, Jennifer L. Clarke, Nancy Ann Oberheim Bush, Shawn L. Hervey-Jumper, Philip Theodosopoulos, Susan M. Chang, Mitchel S. Berger, Hideho Okada

**Affiliations:** 1 Department of Neurological Surgery, University of California, San Francisco, San Francisco, California, USA.; 2 Department of Respiratory Medicine & Rheumatology, Graduate School of Biomedical Sciences, Tokushima University, Tokushima, Japan.; 3 Department of Neurology,; 4 Helen Diller Family Comprehensive Cancer Center, and; 5 Division of Neuropathology, Department of Pathology, University of California, San Francisco, San Francisco, California, USA.; 6 Division of Pediatric Hematology/Oncology, University of Minnesota School of Medicine, Minneapolis, Minnesota, USA.; 7 Oncovir Inc., Washington, DC, USA.; 8 Department of Epidemiology and Biostatistics, University of California, San Francisco, San Francisco, California, USA.; 9 Parker Institute for Cancer Immunotherapy, San Francisco, California, USA.

**Keywords:** Oncology, Vaccines, Brain cancer, Cancer immunotherapy, T cells

## Abstract

**BACKGROUND:**

Long-term prognosis of WHO grade II low-grade gliomas (LGGs) is poor, with a high risk of recurrence and malignant transformation into high-grade gliomas. Given the relatively intact immune system of patients with LGGs and the slow tumor growth rate, vaccines are an attractive treatment strategy.

**METHODS:**

We conducted a pilot study to evaluate the safety and immunological effects of vaccination with GBM6-AD, lysate of an allogeneic glioblastoma stem cell line, with poly-ICLC in patients with LGGs. Patients were randomized to receive the vaccines before surgery (arm 1) or not (arm 2) and all patients received adjuvant vaccines. Coprimary outcomes were to evaluate safety and immune response in the tumor.

**RESULTS:**

A total of 17 eligible patients were enrolled — 9 in arm 1 and 8 in arm 2. This regimen was well tolerated with no regimen-limiting toxicity. Neoadjuvant vaccination induced upregulation of type-1 cytokines and chemokines and increased activated CD8^+^ T cells in peripheral blood. Single-cell RNA/T cell receptor sequencing detected CD8^+^ T cell clones that expanded with effector phenotype and migrated into the tumor microenvironment (TME) in response to neoadjuvant vaccination. Mass cytometric analyses detected increased tissue resident–like CD8^+^ T cells with effector memory phenotype in the TME after the neoadjuvant vaccination.

**CONCLUSION:**

The regimen induced effector CD8^+^ T cell response in peripheral blood and enabled vaccine-reactive CD8^+^ T cells to migrate into the TME. Further refinements of the regimen may have to be integrated into future strategies.

**TRIAL REGISTRATION:**

ClinicalTrials.gov NCT02549833.

**FUNDING:**

NIH (1R35NS105068, 1R21CA233856), Dabbiere Foundation, Parker Institute for Cancer Immunotherapy, and Daiichi Sankyo Foundation of Life Science.

## Introduction

Gliomas are the most common primary malignant CNS tumors (1). As of 2016, they are classified according to histology and molecular characteristics as grade I–IV by the WHO (2). Among these clinically and molecularly diverse tumors, WHO grade II low-grade gliomas (LGGs), which include diffuse astrocytomas and oligodendrogliomas, are more common in adults during the third and fourth decades of life. Secondary to their infiltrative nature, they are not curable with resection (3). Survival is shortened by the tendency for malignant transformation into more aggressive WHO grade III or IV high-grade gliomas (HGGs; refs. 3, 4). Even with multimodal therapy (i.e., surgery, radiation therapy, chemotherapy), their invasive growth and resistance to therapy result in recurrence and death in most patients within 1 to 2 decades of diagnosis (5–7). Furthermore, the majority of LGGs harbor gain-of-function mutations in isocitrate dehydrogenase (IDH) 1 or 2 (8, 9). The oncometabolite D-2-hydroxyglutarate (2-HG) produced by mutant IDH is known to promote glioma genesis and to induce immunosuppressive effects in the tumor microenvironment (TME; refs. 10, 11).

Taken together, an LGG can be considered a premalignant condition for an HGG, such that novel interventions to prevent malignant transformation may improve outcomes. Immunotherapeutic modalities, such as vaccines, may offer a safe and effective option for these patients given their tumors’ slower growth rate (in contrast with HGGs), which should allow sufficient time for multiple immunizations and higher levels of antiglioma immunity. The immune system of patients with LGGs may not be as compromised as that of patients with HGGs because patients with LGGs have demonstrated an excellent immunological response to the vaccines (12). Furthermore, the generally mild toxicity of vaccines may have advantages over chemotherapy or radiation therapy for long-term cognitive and quality-of-life impairments.

To implement effective immunoprevention against recurrence with malignant transformation to an HGG, we employed an allogeneic cell lysate–based vaccine from the glioma stem cell line GBM6-AD, which was isolated from a patient diagnosed with glioblastoma multiforme (GBM; ref. 13). These cells express several glioma-associated immunogenic antigens (GAAs) — e.g., IL-13Rα2, EphA2, and Her-2 — that are frequently expressed at some levels in LGGs and at higher levels in HGGs (12), suggesting that immune responses against GBM6-AD may target existing LGG tissues and provide protective immunity against HGGs. In the initial study in patients with GBM, the autologous DC-based vaccine loaded with GBM6-AD was well tolerated and associated with an immune response in a subset of patients (13).

TLR3 ligands are known to serve as natural inducers of proinflammatory cytokines capable of promoting type-1 adaptive immunity, and TLR3 is abundantly expressed by cells within the CNS (14). We have previously reported that coadministration of a TLR3 ligand, polyinosinic-polycytidylic acid (poly-IC) stabilized with poly-lysine and carboxymethylcellulose (poly-ICLC), enhanced CNS tumor-trafficking of vaccine-induced effector T cells, resulting in a therapeutic effect in rodent CNS tumor models in a CXCL10-dependent manner (14). Our pilot study evaluating the combination of poly-ICLC with a peptide-based vaccine in patients with LGGs demonstrated robust vaccine-specific response and was well tolerated (12).

Finally, the effect of immunotherapy on the TME needs to be evaluated properly. A sampling of recurrent tumors after immunotherapy failure is not ideal because of the inconsistency in the timing of sampling among the patients and the potential for acquired resistance. As such, we conducted a pilot vaccine study to evaluate the safety and immunological effects of vaccination with GBM6-AD lysate and poly-ICLC with presurgical randomization and immunotherapy of patients, allowing for prospective procurement and evaluation of tumor samples.

## Results

### Patient characteristics.

From September 2016 until November 2019, a total of 33 patients were screened, and 8 were excluded for various reasons. Twenty-five patients were randomized — 13 into arm 1 and 12 into arm 2 — and underwent resection. Seventeen patients were available for analysis; 4 patients were excluded after resection secondary to confirmation of malignant transformation; 1 was excluded secondary to insufficient tissue, and 3 withdrew consent (Supplemental Figure 1; supplemental material available online with this article; https://doi.org/10.1172/JCI151239DS1). Nine patients (median age, 43 years; 33.3% female, 66.6% male) were available for analysis from arm 1 and 8 patients (median age, 33 years; 62.5% female, 37.5% male) from arm 2 (Table 1). Only 1 case in each arm was newly diagnosed, and all patients harbored an IDH1 mutation, though this was not required for eligibility, and 1 patient received neoadjuvant vaccine prior to glioma diagnosis. Of the patients randomized to arm 1, 89% had an oligodendroglioma versus 38% with an oligodendroglioma in arm 2. To help account for this bias and delineate the immunological characters of oligodendrogliomas and astrocytomas, we compared the immune cell compositions in these tumor types by CIBERSORTx deconvolution analyses of the RNA-Seq gene expression data from The Cancer Genome Atlas (TCGA) (ref. 15 and Supplemental Figure 2). We found no remarkable differences between oligodendrogliomas and astrocytomas, while the differences between IDH-WT and IDH-mutant gliomas were much more remarkable. Furthermore, we analyzed our clinical data in the current study a) with all eligible patients and b) with only patients who had oligodendrogliomas. There were no differences between arms 1 and 2 regarding race, ethnicity, the median time between surgical resection and first adjuvant vaccination, and the median number of administered adjuvant vaccines.

### Treatment and safety.

**Figure 1:**
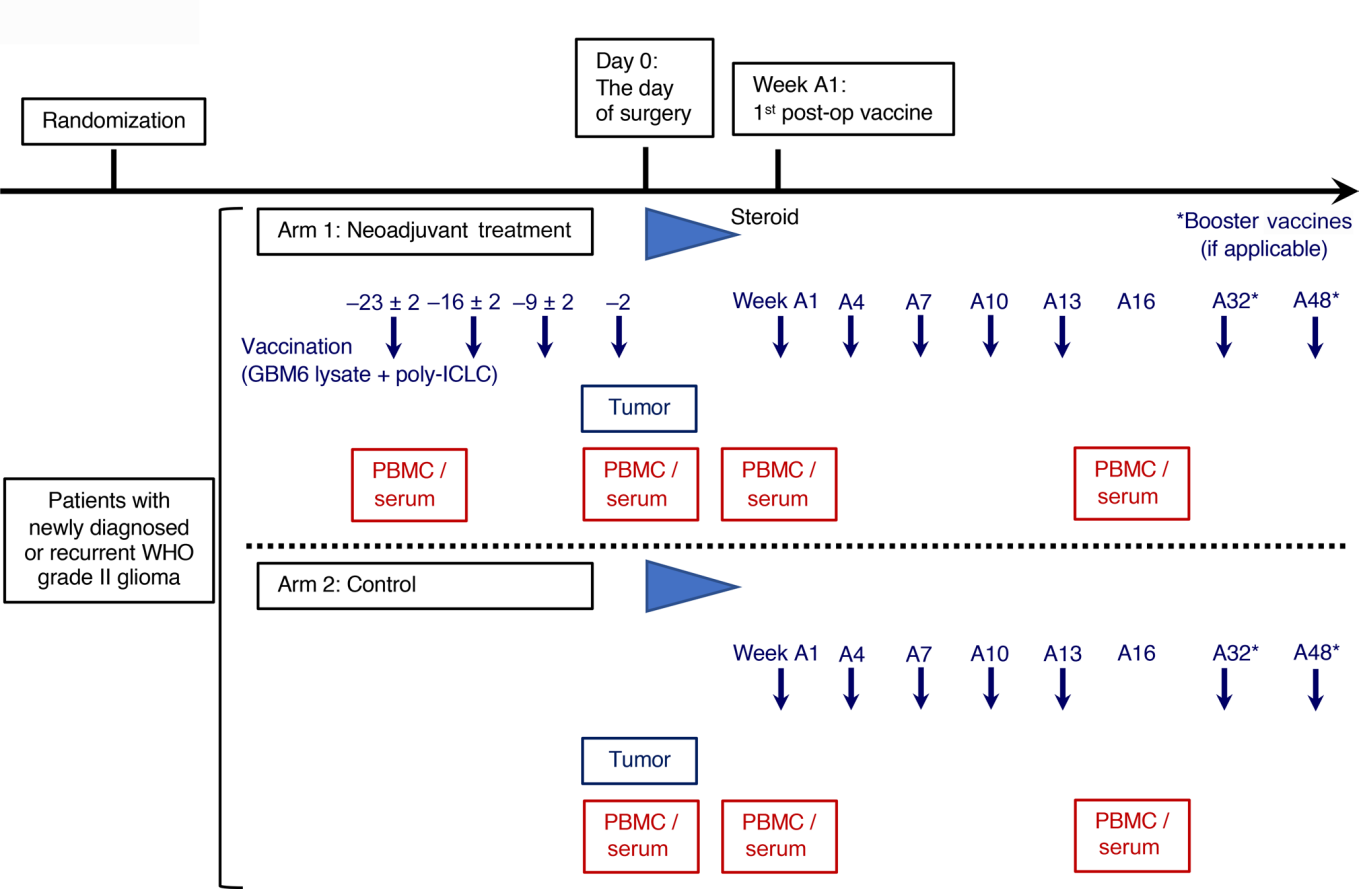
Study schema. Patients were randomized to arm 1 or 2. Patients in arm 1 received GBM6-AD lysate and poly-ICLC on days –23 ± 2, –16 ± 2, –9 ± 2, and –2 relative to the scheduled surgery. At least 2 weeks after the postoperative steroid was tapered, but within 10 weeks after surgery, patients in arm 1 and arm 2 started receiving the GBM6-AD/poly-ICLC vaccines every 3 weeks for 5 doses (weeks A1, A4, A7, A10, and A13; defined as the weeks from first adjuvant vaccine dose) followed by booster vaccines at weeks A32 and A48.

The study design is shown in Figure 1. All arm 1 patients received 4 doses of neoadjuvant vaccination, and most of the patients in both arms completed all scheduled adjuvant vaccinations (Table 1). Treatment was well tolerated with only 1 grade 3 and no grade 4 or 5 treatment-related adverse events (TRAEs). The most common TRAE was injection site reaction (Table 2). No patients experienced any regimen-limiting toxicity.

### Clinical outcomes.

The median time of follow-up for all patients enrolled and who received the A1 vaccine (“A1” is defined as the date of the first adjuvant/postoperative vaccine) was 20.81 months (95% CI 15.2–28.9 months) and all patients remain alive. Progression-free survival (PFS) was calculated from the time of postoperative A1 vaccine to time of centrally confirmed imaging progression per Response Assessment in Neuro-Oncology (RANO) for LGGs (16). Median PFS was 11.0 months (95% CI 10.8–15.4 months) (Supplemental Figure 3A). Event-free survival (EFS) was calculated from the time of the postoperative A1 vaccine to the time of new therapy. Median EFS was 23.7 months (95% CI 19.5–not reached; Supplemental Figure 3B). Of the 6 patients who went on to receive additional treatment, 3 had second surgery: 1 confirmed malignant progression to anaplastic oligodendroglioma and 2 confirmed recurrent LGGs. Of the 3 patients who went on to receive additional treatment without surgery, 1 had evidence of enhancement at the time of recurrence to suggest malignant transformation. There were no significant differences between PFS or EFS between trial arms (Supplemental Figure 3, C and D).

### Neoadjuvant vaccination with GBM6-AD lysate and poly-ICLC induces type-1 cytokines and chemokines in peripheral blood.

**Figure 2:**
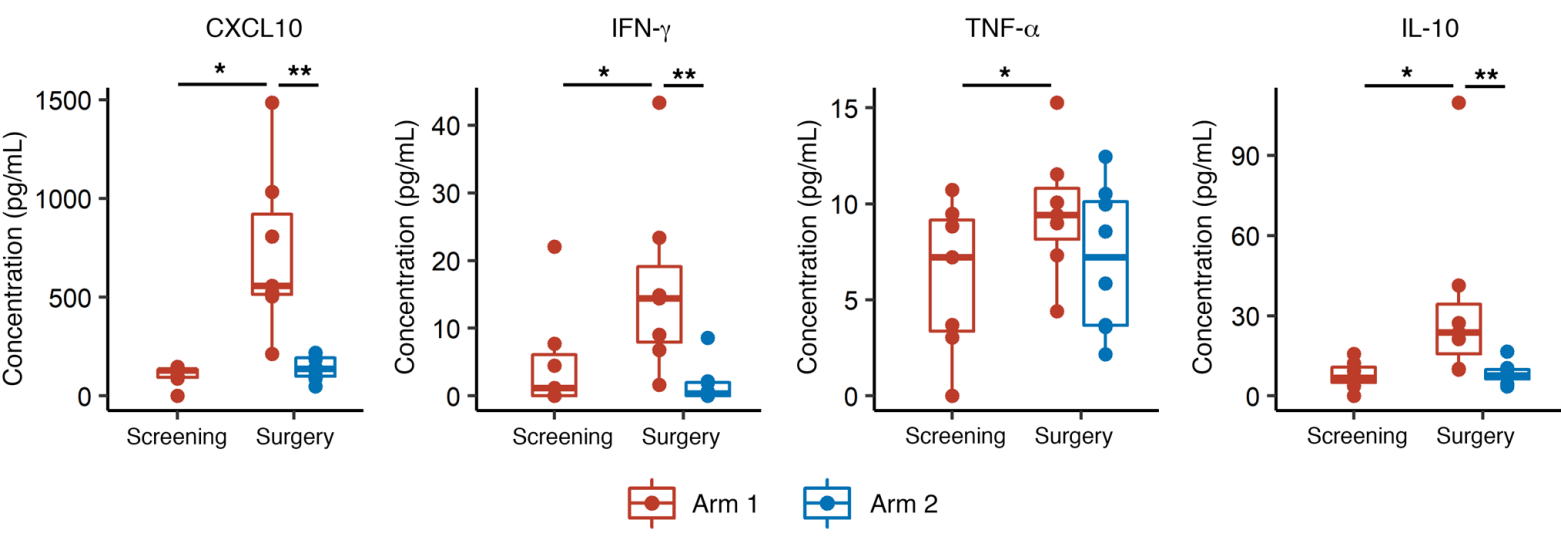
Neoadjuvant vaccinations with GBM6-AD lysate and poly-ICLC induced the upregulation of type-1 chemokines and cytokines in peripheral blood. Serum concentrations of multiple chemokines and cytokines were measured by Luminex multiplex assay. The type-1 chemokine CXCL10 was elevated in arm 1 samples on the day of surgery, within 48 hours of the last neoadjuvant vaccination. Effector cytokines, such as IFN-γ, TNF-α, and IL-10, also demonstrated significant upregulation after the neoadjuvant vaccines. *P < 0.05 (calculated by paired Wilcoxon test) and **P < 0.05 (calculated by nonpaired Wilcoxon test).

To evaluate the immune response induced by the study regimen in peripheral blood, we first performed the Luminex multiplex assay with serum samples to evaluate the induction of cytokines and chemokines. Serum samples were drawn at screening (for arm 1 patients only) on the day of surgery, A1, and A16 (Figure 1). We previously demonstrated that tumor-specific type-1 T cells, which predominantly secrete IFN-γ, can efficiently traffic into CNS tumors and mediate effective therapeutic efficacy via type-1 chemokine CXCL10 (17, 18). We detected significantly elevated serum concentration levels of CXCL10, IFN-γ, TNF-α, and IL-10 in arm 1 patients on the day of surgery, which was within 48 hours after the last dose of the neoadjuvant vaccinations, compared with arm 1 samples at screening or arm 2 samples on the day of surgery (Figure 2). On the other hand, in the serum samples at A16, which was 3 weeks after the final adjuvant vaccination (A13), there was no upregulation in either group (Supplemental Figure 4A), suggesting a short window for cytokine response in peripheral blood, which is consistent with our previous results (17). We observed the same trends when we selectively analyzed the data from oligodendroglioma patients, though the differences in IFN-γ or TNF-α concentration between screening and day of surgery time points in arm 1 patients only achieved borderline significance because of the smaller number of cases (*P =* 0.06, Supplemental Figure 4B).

### PD-1^+^GZMB^hi^Tbet^hi^ effector memory and GZMB^hi^Tbet^hi^ effector CD8^+^ T cells increase after neoadjuvant vaccination.

**Figure 3:**
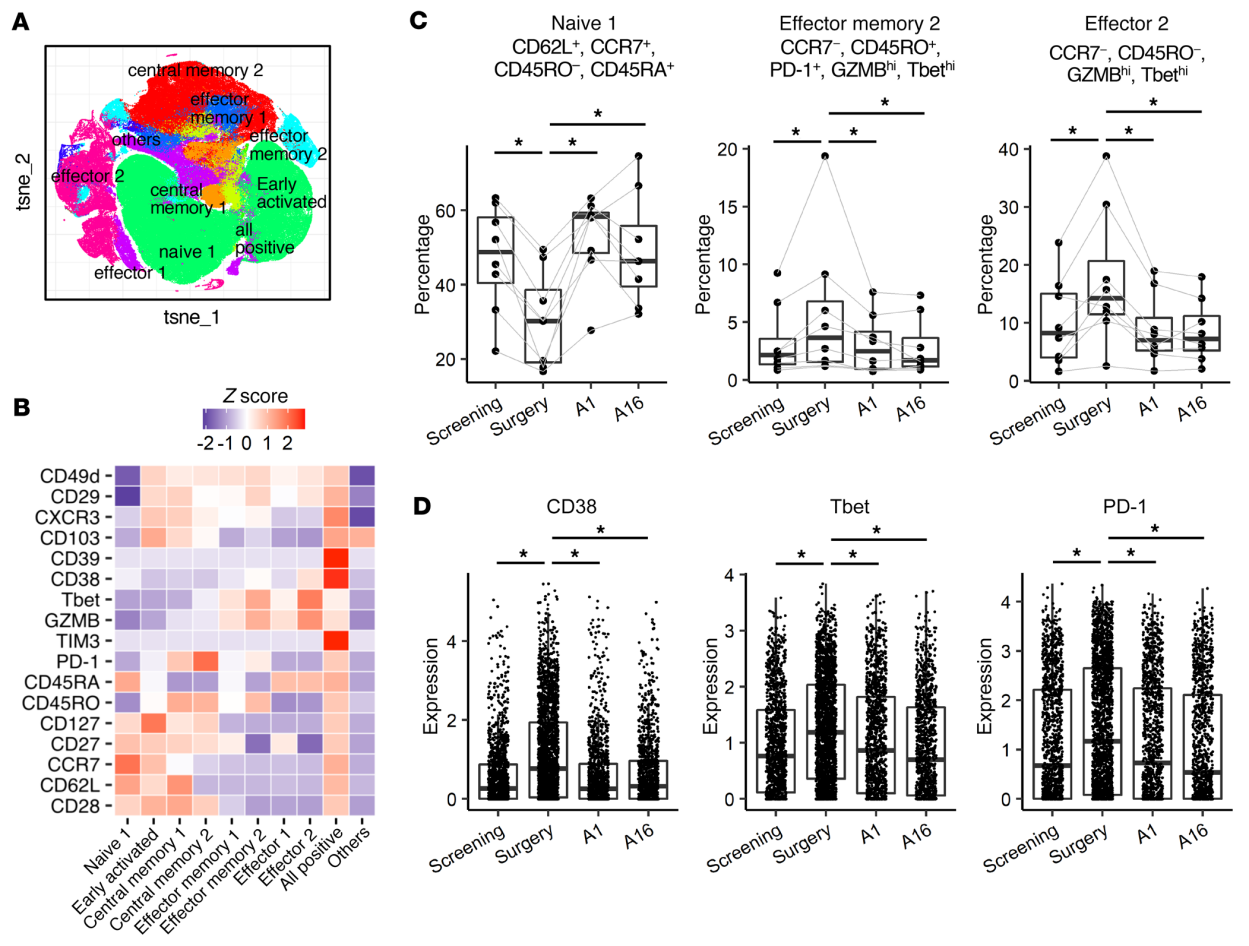
Mass cytometric analyses detected increases of PD-1^+^GZMB^hi^Tbet^hi^ effector memory and GZMB^hi^Tbet^hi^ effector CD8^+^ T cells after the neoadjuvant vaccines. (**A**) T-distributed stochastic neighbor embedding (t-SNE) plot of CD8^+^ T cells. To evaluate the vaccine-induced changes of phenotype in peripheral blood, mass cytometric analyses were conducted. CD8^+^ T cells were subjected to dimension reductional algorithm t-SNE for visualization in 2D space and clustered by FlowSOM based on the expression status of 7 differentiation markers (CD62L, CD27, CD127, CCR7, CD45RO, CD45RA, and PD-1). (**B**) Heatmap visualizing the relative expression (*z* score) of T cell–relevant markers in each subpopulation. Each cluster was annotated based on the expression status of differentiation markers as listed above. (**C**) The longitudinal analyses of proportions of each subpopulation in arm 1 patients. Neoadjuvant vaccination with GBM6-AD and poly-ICLC increased PD-1^+^GZMB^hi^Tbet^hi^ effector memory and GZMB^hi^Tbet^hi^ effector CD8^+^ T cells while decreasing naive CD8^+^ T cells. *P < 0.05 (paired Wilcoxon test). (**D**) The expression levels of activation markers, such as CD38, Tbet, and PD-1, on the PD-1^+^GZMB^hi^Tbet^hi^ effector memory cells were enhanced in the samples obtained after the neoadjuvant vaccines. *P < 0.05 (nonpaired, 2-tailed *t* test).

To evaluate the regimen-induced changes of phenotype in PBMCs, we conducted mass cytometric analyses from samples collected at screening (in arm 1 only), day of surgery, A1, and A16. We first extracted CD8^+^ T cells using a conventional CD8^+^ gating strategy (Supplemental Figure 5). CD8^+^ T cells were clustered on the t-distributed stochastic neighbor embedding (t-SNE) plot and grouped into 10 subpopulations and annotated based on the expression status of differentiation markers, such as CD62L, CD27, CD127, CCR7, CD45RO, and CD45RA (Figure 3, A and B). By analyzing the proportions of each subpopulation among CD8^+^ T cells, we found that PD-1^+^Granzyme B^hi^(GZMB^hi^)Tbet^hi^ effector memory and GZMB^hi^Tbet^hi^ effector CD8^+^ T cells were upregulated at surgery, whereas naive CD8^+^ T cells were downregulated in arm 1 patients (Figure 3C). We found that some activation markers, such as CD38, Tbet, and PD-1, were expressed on all cells in this effector memory cluster and upregulated after neoadjuvant vaccination (Figure 3D). However, we did not observe any significant differences when directly comparing the proportions of these subpopulations between arm 1 and arm 2 (Supplemental Figure 6). The same trend was observed in the patients with oligodendroglioma (Supplemental Figure 7). These results indicate that the study regimen induced type-1 immune responses in peripheral blood.

We also evaluated associations between vaccine-induced immune responses and clinical outcomes. Among arm 1 patients, we defined an immunological responder as a patient who demonstrated a 10% or higher increase in the proportion of either PD-1^+^GZMB^hi^Tbet^hi^ effector memory or GZMB^hi^Tbet^hi^ effector population after neoadjuvant vaccination. This led to identification of 4 patients (with oligodendrogliomas; patients 103-018, -026, -29, -51) as immunological responders. There were no clear associations between the immunological response and PFS (Supplemental Figure 8).

### Vaccine-reactive CD8^+^ T cells with effector phenotype migrate into the TME.

**Figure 4:**
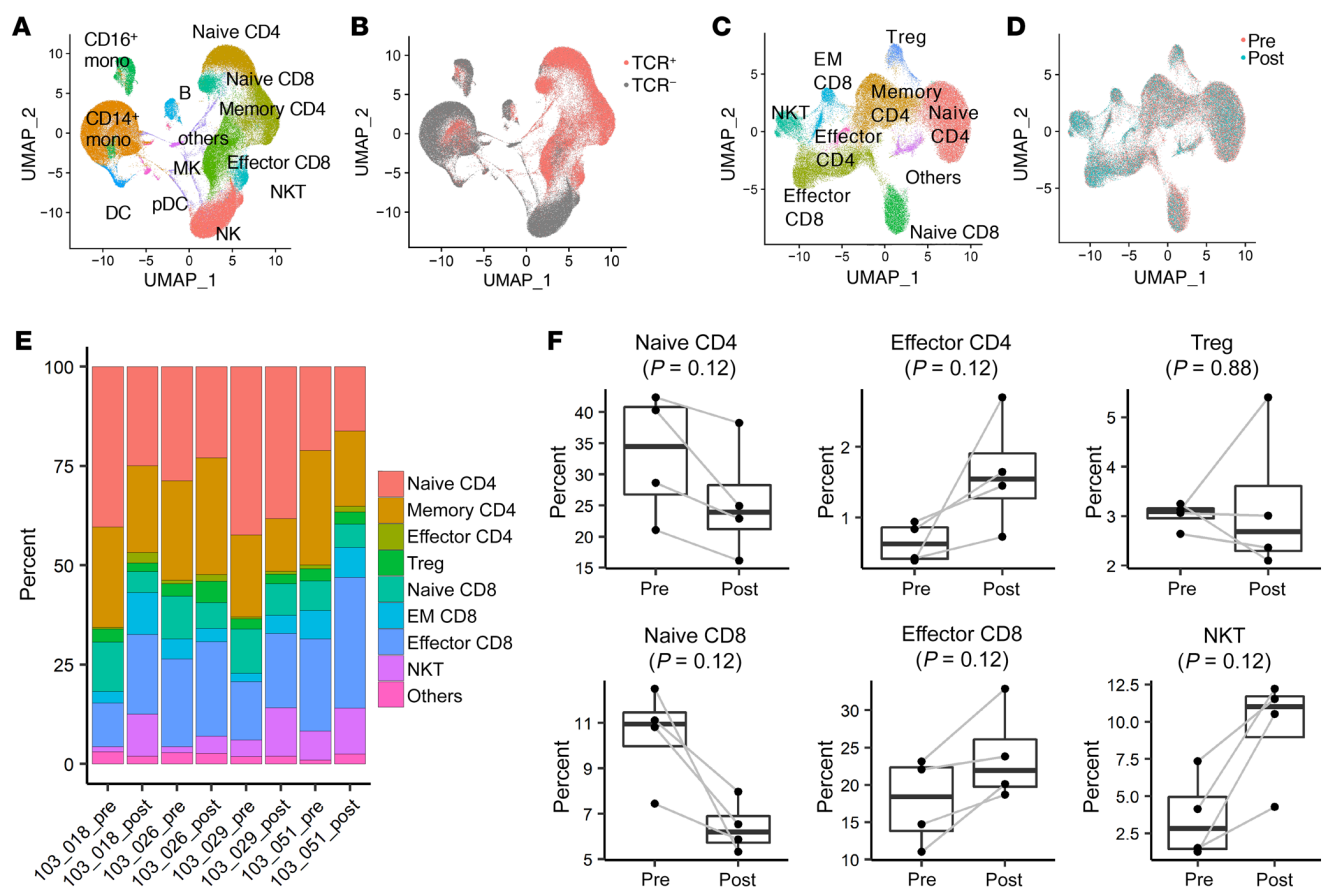
scRNA-Seq analyses revealed the increases of effector CD4^+^ and CD8^+^ and decreases of naive CD4^+^ and CD8^+^ T cell populations after the neoadjuvant vaccinations. ScRNA-Seq and scTCR-Seq analyses on the 10x Genomics platform were conducted in PBMCs obtained from the 4 immunological responders (patients 103-018, -26, -29, -51) at baseline and after neoadjuvant vaccines. (**A**) UMAP of pooled PBMCs from all 4 patients at baseline and after neoadjuvant vaccines. Clusters were annotated based on the expression of known marker genes. Mono, monocyte; pDC, plasmacytoid DC; MK, megakaryocyte; B, B cells. (**B**) UMAP was colored by TCR detection. TCRs were mainly detected in 5 clusters that represent T cell and NKT cell populations (pink). (**C**) UMAP of T cells and NKT cells. T cell and NKT cell populations were reclustered and grouped into 9 subpopulations. EM, effector memory. (**D**) UMAP of T cells and NKT cells was colored by treatment status (either pre- or post-vaccination). Cytotoxic T cells, such as effector CD8^+^ T cells and NKT cells, were enriched in postvaccinated samples (light blue). (**E**) The bar plot showing the proportion of each cell cluster in each sample. (**F**) Quantification of each cell cluster in prevaccinated and postvaccinated samples. The proportion of effector T cells showed a trend toward an increase in postvaccinated samples while that of naive T cells showed a trend toward a decrease. *P* values were calculated by paired Wilcoxon test.

To characterize the gene expression, subset proportions, and T cell receptor (TCR) profile of T cells in PBMCs, we analyzed pre- and post-neoadjuvant vaccinated PBMCs from 4 immunological responders (patients 103-018, -26, -29, -51), using droplet-based 5′ single-cell RNA-Seq (scRNA-Seq) and single-cell TCR-Seq (scTCR-Seq) with the 10x Genomics platform. We obtained scRNA-Seq profiles from a total of 154,929 PBMCs with paired TCR sequences in 63,932 out of 76,432 T cells (83.6%). We identified 17 cell clusters based on scRNA-Seq profiles (Figure 4A) and confirmed TCR-α and -β sequences in 5 T cell and NKT cell clusters (Figure 4B). We then reclustered these T cells and NKT cells into 9 populations based on their gene expression profiles (Figure 4C and Supplemental Figure 9A). When comparing prevaccination versus postvaccination cytotoxic T cells, effector CD8^+^ T cells and NKT cells were enriched in postvaccinated samples (Figure 4D and Supplemental Figure 9B). In postvaccinated cells, there was an increased proportion of effector CD4 and CD8 cells and decreased proportion of naive CD4 and CD8 populations, consistent with results from the mass cytometry analysis (Figure 4, E and F).

**Figure 5:**
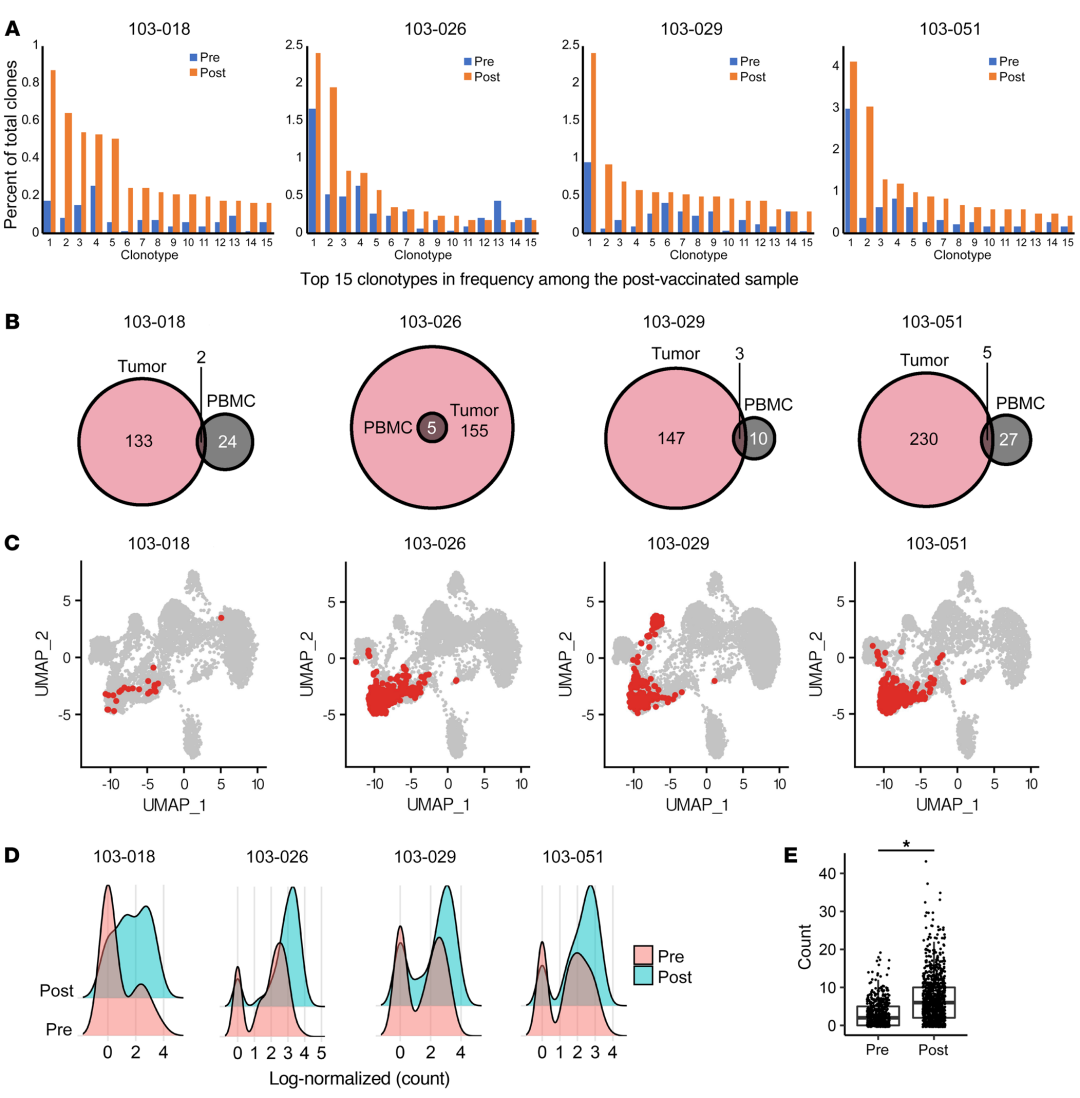
Vaccine-reactive CD8^+^ T cell clones with an effector phenotype migrated into the tumor microenvironment. (**A**) The top 15 frequent clonotypes in postvaccinated samples were extracted, and their frequencies were compared. Most of these clonotypes showed higher frequencies in postvaccinated samples than at baseline. (**B**) The TCR clonotypes that were enriched in postvaccinated PBMCs were extracted with an adjusted *P* value less than 0.15. Patients 103-018, -26, -29, and -51 were found to have 26, 5, 13, and 32 enriched TCR-b clonotypes, respectively, in their PBMCs. Some of these clonotypes were also found in the TCR repertoire of corresponding tumors (determined by bulk TCR-Seq). (**C**) The T cell clones that had these overlapped clonotypes mostly belonged to the effector CD8 cluster in PBMCs in all cases. (**D** and **E**) The expression of GZMB was upregulated by neoadjuvant vaccinations in these T cell clones. Log-normalized (count) on *x* axis was calculated as log (count/[total count of the cell] × 10,000 + 1). *P < 0.05 (nonpaired, 2-tailed *t* test).

Data from scTCR-Seq identified T cell clones that expanded in postvaccination PBMCs. When we focused on the top 15 frequent clonotypes in postvaccinated samples, we found them to be enriched in postvaccine samples compared with screening (Figure 5A). By extracting the TCR clonotypes enriched in the postvaccine samples with adjusted *P* value less than 0.15, we identified 26, 5, 13, and 32 enriched TCR-β sequences in patients 103-018, -26, -29, and -51, respectively (Figure 5B). We also performed bulk TCR-Seq using genomic DNA from paired resected tumor specimens using ImmunoSEQ and found that some TCR-β clonotypes enriched in postvaccinated peripheral blood were also identified in the corresponding tumor tissue (Figure 5B). The T cell clones from these shared clonotypes were from the effector CD8 cluster in the PBMCs (Figure 5C) and GZMB expression was upregulated in these postvaccine samples (Figure 5, D and E). Moreover, within the effector CD8 cluster, the T cells with shared clonotypes expressed a higher level of GZMB than other T cells (Supplemental Figure 10), suggesting that some of the vaccine-reactive T cells with cytotoxic CD8^+^ phenotype migrated into the TME. To address whether the patients’ T cell responses were directed against the vaccine-derived antigens, we conducted further evaluations of the CD8^+^ T cells derived from these immunological responders at the surgery time point (after neoadjuvant vaccinations). GBM6-AD cells express some of the well-characterized GAAs, such as EphA2 and IL-13Rα2 (Supplemental Figure 11A). Per RNA-Seq, both GBM6-AD and patient-derived LGG tumors expressed these antigens, albeit at different levels (Supplemental Figure 11B). We then stimulated those T cells with autologous DCs pulsed with either GBM6-AD lysate or recombinant protein for EphA2 and IL-13Rα2 and evaluated whether the TCR clones that had expanded after the vaccines in PBMCs could further expand in response to the in vitro stimulations. In 1 immunological responder (patient 103-018), 4 of the top 15 most abundant CD8^+^ T cell TCR clonotypes (Supplemental Figure 12A; clones 1, 3, 7, and 9) enriched more than 20% in response to the GAA stimulation when compared with the control group with no stimulation. However, we were not able to demonstrate a robust response of these TCR clones to GBM6-AD lysate (Supplemental Figure 12A). Nevertheless, scTCR-Seq analyses showed that the frequencies of these 4 CD8^+^ T cell clones among total T cells in PBMCs increased after the vaccination with GBM6-AD in this patient (Supplemental Figure 12B). We also confirmed that none of these TCR clonotypes matched the known viral antigen–specific TCRs that are listed in the VDJdb database (https://vdjdb.cdr3.net/). These results suggest that at least some of the TCR clones responded to the vaccine-derived antigens.

### The proportion of PD-1^+^CXCR3^hi^ effector memory CD8^+^ T cells was significantly higher in the vaccinated TME.

**Figure 6:**
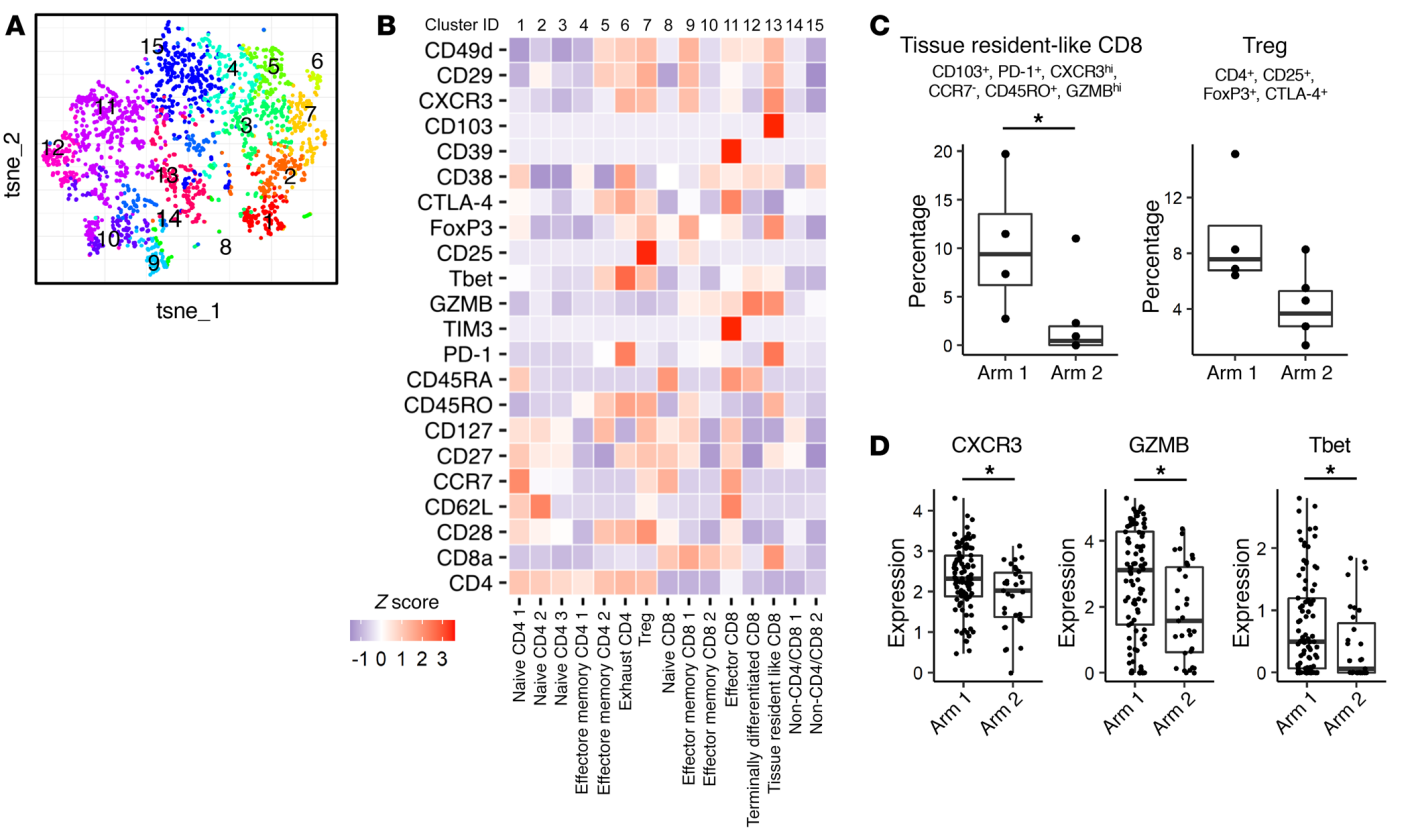
The proportion of tissue resident–like CD8^+^ T cells with effector memory phenotype was significantly higher in the vaccinated tumor microenvironment. Single-cell suspensions dissociated from tumor samples from arm 1 (4 cases) and arm 2 (6 cases) were analyzed by mass cytometry. (**A**) CD3^+^ T cells were subjected to dimension reductional algorithm t-SNE and clustered by FlowSOM based on the expression status of 10 differentiation markers (CD4, CD8a, CD62L, CD27, CD127, CCR7, CD45RO, CD45RA, CD25, and PD-1). (**B**) Heatmap visualizing the relative expression (*z* score) of T cell–relevant markers in each subpopulation. Each cluster was annotated based on the expression status of differentiation markers as listed above. (**C**) The proportion of tissue resident–like CD8^+^ T cells with effector memory phenotype (CD103^+^, PD-1^+^, CXCR3^hi^, CCR7^–^, CD45RO^+^, GZMB^hi^) was significantly higher in arm 1 samples. *P < 0.05 (nonpaired Wilcoxon test). The proportion of Tregs in arm 1 showed a trend toward a higher percentage than arm 2 but without statistical significance. (**D**) TILs in this tissue resident–like CD8^+^ T cell cluster in arm 1 tumors demonstrated significantly higher expression levels for the CXCL10 receptor CXCR3, GZMB, and Tbet than those in arm 2 tumors.

We analyzed the immune profile of tumor-infiltrating lymphocytes (TILs) by mass cytometry. Four samples from arm 1 and 6 from arm 2 were available for this analysis. Because of the low frequency of leukocytes in the resected tumor samples, we extracted CD3^+^ T cells instead of CD8^+^ T cells (Supplemental Figure 13) and subgrouped these cells into 15 clusters (Figure 6, A and B). The proportion of CD103^+^CD8^+^ T cells with an effector memory phenotype (19) was significantly higher in arm 1 tumors (Figure 6C). These T cells were also highly positive for the CXCL10 receptor CXCR3. The proportion of Tregs in arm 1 trended higher but was not statistically significant (Figure 6C). Among the tissue resident–like CD8^+^ T cell cluster, TILs in arm 1 tumors demonstrated significantly higher expression levels for CXCR3, GZMB, and Tbet (Figure 6D). When we evaluated only oligodendrogliomas (3 cases in each arm), TILs in arm 1 trended toward a higher proportion of CD103^+^CXCR3^hi^ tissue resident–like CD8^+^ T cells and effector CD8^+^ T cell populations but without statistical significance (Supplemental Figure 14). These results suggest the possibility that CXCR3^+^CD8^+^ T cells were recruited into the TME by the CXCL10/CXCR3 axis and differentiated to tissue resident–like cells by the neoadjuvant vaccine. However, we were unable to detect any significant difference in expression of immune-related genes in the tumors derived from arm 1 versus arm 2 patients using bulk RNA-Seq analyses (Supplemental Figure 15).

## Discussion

Despite the unmet need for developing effective and safe therapy for WHO grade II LGGs, immunotherapy has not been extensively investigated in this population (12). By implementing a neoadjuvant design, we prospectively evaluated the impact of the GBM6-AD lysate vaccine with poly-ICLC in the peripheral blood and TME. Although the neoadjuvant vaccination–induced systemic immune response was detectable in peripheral blood, we did not observe an increase of TILs by mass cytometry (Supplemental Figure 13) or remarkable immune responses, such as effector molecule or chemokine productions, based on bulk RNA-Seq analysis of the resected tumor tissues (Supplemental Figure 15). Divergence of the response between the peripheral blood and the TME suggests a substantial barrier for the systemic immune response to adequately manifest in the TME. In contrast to the current study, our prior DC-based vaccine study in combination with poly-ICLC detected induction of CXCL10 in GBM samples that recurred after vaccination (17). Gliomas, especially LGGs, have a low degree of T cell infiltration when compared with a variety of other cancers (20), and the blood-brain barrier appears to be more intact in LGGs than in HGGs (21).

However, we were able to detect the presence of vaccine-reactive CD8^+^ T cell clones in the TME (Figure 5, B and C) and increased frequency of activated CD103^+^CXCR3^hi^CD8^+^ T cells in the TME by single cell–based analyses after the neoadjuvant vaccination (Figure 6C). These high-resolution analyses allowed us to detect some impact of the peripherally administered vaccination on the LGG TME. Taken together, future studies will have to integrate more effective strategies to render the LGG TME more permissive to the immune response. Because tumor cells produce a variety of immunoregulatory factors (22), such as TGF-β, combination regimens with blockade of immunosuppressive pathways should be considered.

In the peripheral blood analysis, we detected a clear immune response only on the day of surgery (after neoadjuvant vaccination) in arm 1, but not on A16 (after adjuvant vaccination) in either arm (Figures 2 and 3 and Supplemental Figure 4). This may partially be secondary to differences in frequency of vaccine administration (neoadjuvant and adjuvant vaccines were administered weekly and every 3 weeks, respectively). It may also be due to the interval between the most recent vaccine administration and blood collection (2 days in the neoadjuvant vaccine; 3 weeks in the adjuvant vaccine). We previously reported that a DC-based peptide vaccine with poly-ICLC induced a robust upregulation of CXCL10 that peaked at 24 hours after the first vaccination in peripheral blood (17). Therefore, it is not straightforward to compare the immunological effects of neoadjuvant and adjuvant vaccines directly in the current analyses.

Although we did not observe upregulated IFN-γ signatures in the postvaccine LGG tumors compared with the control LGG tumors (Supplemental Figure 15), recent neoadjuvant immunotherapy trials evaluating anti–PD-1 antibodies in patients with HGG have reported upregulated IFN-γ–related genes (23, 24). Cloughesy et al. showed that neoadjuvant immunotherapy treatment in recurrent GBM was associated with prolonged overall survival (23). The lack of upregulated IFN-γ signatures in the current study may reflect the difference of the immunotherapy regimen and lesser immune-permissive characteristics of IDH-mutant LGGs compared with HGGs (25, 26).

GBM6-AD cells express stem cell markers and some of the GAAs, such as EphA2 and IL-13Rα2 (Supplemental Figure 11), which have defined HLA-binding T cell epitopes in the context of HLA-A*02:01 (27–29). However, because our eligibility criteria did not require HLA typing of individual patients, it was not feasible to identify T cell epitopes included in the GBM6-AD lysate for each patient’s unique HLA type. Nevertheless, our scRNA/TCR-Seq analysis enabled us to identify TCR clonotypes that expanded after the vaccinations and existed in the LGG TME (Figure 5, B and C). None of the expanded TCR clonotypes matched the known viral antigen–specific TCRs. Furthermore, some of the TCR clones that had expanded after the GBM6-AD vaccine in vivo further expanded in response to stimulations with recombinant protein for EphA2 and IL-13Rα2 in vitro (Supplemental Figure 12). These results suggest that at least some of the TCR clones responded to the vaccine-derived GAAs and the immune response elicited by the GBM6-AD is relevant to the immunogenicity of the patient-derived gliomas. However, we observed the increase of TCR clones in response to GAAs in only 1 patient among the 4 immunological responders. The absence of detectable responses in the other patients could be due to the assay sensitivity and different levels of GAA epitope-presentation on a variety of HLA types in those patients. Also, immunosuppressive factors that are expressed by glioma cells, such as CD200 (30), may have suppressed the T cell responses against the GBM6-AD lysate. Moertel et al recently implemented a phase I clinical trial to evaluate the effects of CD200 blockade in the GBM6-AD lysate vaccine in patients with recurrent HGGs (ClinicalTrials.gov NCT04642937). To improve the design of our future vaccine studies, the use of novel algorithms developed for the identification of target epitopes recognized by the TCR repertoire (31, 32) may allow us to identify the antigenic targets.

We recognize several limitations of the current study. First, this is a pilot study with a small sample size, and the follow-up periods have been insufficient to evaluate clinical benefits in patients with LGGs, even preliminarily. Second, the amount of resected tumor available was limited to allow a more extensive analysis, such as scRNA/TCR-Seq of TILs. Third, the histological types (oligodendroglioma vs. astrocytoma) were not adequately balanced in patient assignments between arms 1 and 2. Because of the small sample size, our randomization was based solely on whether the patient was newly diagnosed or recurrent but not on any other factors such as histological types, prior treatment, or time from diagnosis. Oligodendroglioma has a better prognosis than astrocytoma (33). However, to the best of our knowledge, no reports have shown significant immunological differences between these 2 histological types. Our analyses demonstrated that there were no remarkable differences in the immune cell composition between oligodendrogliomas and astrocytomas, although the densities of some immune cells, such as CD4 memory resting cells and follicular helper T cells, showed slight differences (Supplemental Figure 2). On the other hand, the differences between IDH-WT and IDH-mutant gliomas were more remarkable. Further, to evaluate the impact of the vaccine regimen within the same histology, we analyzed data from oligodendroglioma cases alone and observed overall consistent results, suggesting our findings may not be heavily biased on the histology. Fourth, we analyzed the TCR repertoire profiles by scRNA/TCR-Seq only in immunological responders. Additional analyses in nonresponders might help us to better understand the differences in response. Fifth, we treated all patients with the combination of GBM6-AD lysate and poly-ICLC but did not include a group with monotherapy with GBM6-AD vaccine alone or poly-ICLC alone because of the small sample size, although our preclinical data demonstrating the effects of the combination (14) provided a rationale for the design of the current study.

Because the majority of patients with LGGs experience malignant transformation to HGGs (3), we aimed for patients to mount preemptive immunity against antigens expressed in HGGs by vaccinating with an allogeneic glioblastoma-derived GBM6-AD lysate. Cancer immunoprevention is based on the hypothesis that a functioning immune system controls tumor onset and development. Prophylactic vaccines against virally caused cancers have been utilized in the clinic (34). For tumors without viral etiologies, vaccines targeting cancer-associated mucin 1 (MUC1) have been evaluated in patients with colon adenoma as a premalignant disease or resected non–small cell lung cancer (35, 36). Additional analyses will be required to determine whether vaccination with GBM6-AD cell lysate in patients with LGGs would have the potential of immunoprevention for malignant transformation to HGGs. Further, collaborative approaches among investigators who are committed to developing immunoprevention approaches for patients with premalignant diseases would facilitate the advancement of the field.

In conclusion, the current pilot neoadjuvant vaccine study demonstrated that some of the vaccine-reactive CD8^+^ T cells can traffic to the LGG TME, although further refinements of the regimen and more active disruption of the blood-brain barrier may have to be integrated into future immunotherapeutic strategies to achieve a better clinical outcome.

## Methods

### Study design and patients.

This study (ClinicalTrials.gov NCT02549833) is a pilot trial assessing the safety and immunoreactivity of s.c. administration of GBM6-AD lysate in combination with poly-ICLC (Hiltonol, Oncovir) in patients 18 years and older with newly diagnosed or recurrent WHO grade II gliomas (defined as an astrocytoma or oligodendroglioma). Presence of IDH mutation was not a predefined eligibility criteria because the trial was designed prior to the WHO 2016 classification system. Key eligibility criteria included Karnofsky performance status of 70 or greater; the presence of supratentorial, nonenhancing T2-FLAIR lesions; anticipation of at least 500 mg tumor tissue at resection; and no history or clinical suspicion of immune system abnormalities. Prior radiation therapy, chemotherapy, or molecularly targeted therapy were allowed. Patients must have been off corticosteroid for at least 2 weeks before the first neoadjuvant vaccine or adjuvant vaccine.

The study design and flow diagram are summarized in Figure 1 and Supplemental Figure 1, respectively. In brief, after providing consent, patients were randomized to arm 1 or 2. Arm 1 (neoadjuvant vaccination group) patients received GBM6-AD lysate and poly-ICLC (s.c.) on days –23 ± 2, –16 ± 2, –9 ± 2, and –2 relative to the scheduled surgery. At least 2 weeks after the postoperative steroid was tapered, but within 10 weeks after surgery, the GBM6-AD/poly-ICLC vaccines were given and repeated every 3 weeks for 5 doses (weeks A1, A4, A7, A10, and A13; defined as the weeks from first adjuvant vaccine dose) followed by booster vaccines at weeks A32 and A48. Two arm 2 (control group) patients received no vaccine prior to surgery and only received adjuvant vaccination in the same way as arm 1 patients. The randomization took place in a 1:1 ratio between the 2 arms, stratified by newly diagnosed versus recurrent.

Blood samples were obtained on days –23 ± 2 (only arm 1 patients), on the day of surgery, A1, A10, A16, and A32/48 if applicable. MRI was taken at screening (within 28 days prior to study enrollment and randomization), preoperatively (24–48 hours prior to surgery), postoperatively (within 14 days of surgery and 28 days prior to A1 vaccine), A16, and A32/48 if applicable.

The coprimary endpoints of this study are a) safety (the incidence and severity of adverse events associated with the treatment regimen, with an early stopping rule based on the frequency of regimen-limiting toxicities); and b) detection of the vaccine-induced immune response in the resected tumor.

### Follow-up.

All patients were followed for response and toxicity assessments until disease progression, the start of a new therapy, or for a maximum of 18 months from study registration (whichever occurs earlier). Toxicity was determined using the revised National Cancer Institute’s Common Toxicity Criteria version 5.0 for Toxicity and Adverse Event Reporting (CTCAE). Regimen-limiting toxicities were defined as grade 2 or more bronchospasm or generalized urticarial; grade 2 or more allergic reaction; grade 2 or more autoimmune disease; any grade 3 toxicity related to the vaccine, such as grade 3 injection site reaction, hematological or hepatic toxicity, or neurotoxicity.

### Vaccine formulation with GBM6-AD lysate and poly-ICLC.

GBM6-AD lysate was prepared in batches by the University of Minnesota Molecular and Cellular Therapeutics Facility using the established allogeneic glioblastoma stem cell line GBM6-AD as the antigen source, as previously described (13). Dose vials were made under Good Manufacturing Practice (GMP) conditions for administration under Investigational New Drug (IND) 16,794 and provided by David McKenna Jr. at University of Minnesota. The lysate was supplied in vials each containing 0.5 mL solution with a concentration of 2 mg/mL and stored in liquid nitrogen. Poly-ICLC, a synthetic complex of polyinosinic and polycytidylic acid, stabilized with polylysine and carboxymethyl cellulose, was available from Oncovir, Inc. It was supplied in vials each containing 1 mL of translucent solution with a concentration of 2 mg/mL and stored in a refrigerator. On the day of the scheduled vaccine, GBM6-AD lysate (1 mg protein in 0.5 mL) was mixed with 0.7 mL (1.4 mg) poly-ICLC to formulate a dose for s.c. administration.

### Processing of human samples.

Patients’ PBMCs and serum were isolated by density gradient centrifugation with Ficoll-Paque (GE Healthcare) and cryopreserved for further analysis. The freshly resected tumor tissue was minced using scalpels and digested (3 mg/mL collagenase IV, 1 mg/mL DNase, and 2 mg/mL trypsin inhibitor soybean in PBS) at 37°C for 30 minutes using a shaking heater. The samples were then filtered through a 70 mm cell strainer and washed twice with PBS. The cells were then cryopreserved for further analysis.

### Luminex multiplex assay.

Cytokine and chemokine analyses by multiplex assay were performed by the Immune Assessment Core at University of California, Los Angeles (UCLA). A MILLIPLEX human magnetic bead kit with a panel of 38 analytes (EMD Millipore, HCYTMAG-60K-PX38) was used per the manufacturer’s instructions on a DropArray 96-well plate (Curiox). Briefly, 5 mL undiluted human serum samples were mixed with 5 mL magnetic beads and allowed to incubate overnight at 4°C while shaking. After washing the plates 3 times with wash buffer (PBS with 0.1% BSA and 0.05% Tween 20) in a DropArray LT Washing Station MX96 (Curiox), 5 mL of detection antibody was added and incubated for 1 hour at room temperature. Next, 5 mL streptavidin-phycoerythrin conjugate was added to the reaction mixture and incubated for another 30 minutes at room temperature. After 3 washes, beads were resuspended in sheath fluid, and fluorescence was quantified using a Luminex 200 instrument. Data were analyzed using MILLIPLEX Analyst 5.1 software.

### Mass cytometry data acquisition.

Cryopreserved patient-derived PBMCs or tumor dissociated cells were thawed 1:10 in thawing media (2% human AB serum containing X-VIVO + 25 U/mL Benzonase). Cells were incubated in 5 mM of cisplatin (Cell-ID Cisplatin; Fluidigm), allowing for the distinguishing of live cells. PBMCs, but not tumor dissociated cells, were then fixed with 1.6% PFA and barcoded with Cell-ID 20-Plex Pd Barcoding Kit (Fluidigm). After Fc blocking (human TruStain FcX; BioLegend), cells were stained with metal-conjugated surface antibody cocktail (Supplemental Table 1). Cells were then permeabilized with Perm-S buffer (Fluidigm) and stained with intracellular antibody cocktail (Supplemental Table 1), followed by resuspension in Iridium intercalator (Cell-ID Intercalator; Fluidigm) solution overnight. Cells were then washed and resuspended in running buffer consisting of a 1:10 dilution of normalization beads (EQ Four Element Calibration Beads; Fluidigm) in deionized water. Samples were then acquired on the Fluidigm Helios Mass Cytometer and resultant data was exported to FCS files for further processing. In the PBMC analyses, 8 samples (samples from 4 time points from 2 patients) were barcoded, stained, and acquired on a mass cytometer simultaneously in each experiment.

### Processing of mass cytometry data.

To control for sensitivity variability of the Helios mass cytometer both within and across samples, raw FCS files were processed by the normalizer function provided by the Parker Institute of Cancer Immunotherapy Premessa package on R Studio. Normalization beads were removed on the same platform. The processed files were uploaded to the Cytobank platform and de-barcoded manually. Each immune subpopulation, such as CD8^+^ T cells, was gated and exported as shown in Supplemental Figures 5 and 13. These exported files were then uploaded to the Cytofkit package (37), where immune cells were subjected to dimension reductional algorithm t-SNE for visualization in 2D space and clustered using FlowSOM. The cells in each cluster were then phenotyped and analyzed using *z* score–normalized marker expression and population data, respectively. All analytic outputs were generated on R Studio unless noted otherwise.

### ScRNA-Seq and scTCR-Seq.

Preparation of scRNA-Seq and scTCR-Seq libraries and sequencing were performed by CoLabs at University of California San Francisco (UCSF). Chromium Next GEM Single Cell V(D)J Reagent Kit v1.1 (10x Genomics) was used per the manufacturer’s instructions. In brief, cryopreserved patient-derived PBMCs were washed with PBS containing 0.04% BSA and resuspended in PBS containing 0.04% BSA to a final concentration of 1000 cells per mL. Cells were captured in droplets and nanoliter-scale gel beads-in-emulsion (GEMs) were generated. After reverse transcription and cell barcoding, GEMs were broken, and cDNA was purified using Silane magnetic beads followed by amplification via PCR (98°C for 45 seconds; 13–18 cycles of 98°C for 20 seconds, 67°C for 30 seconds, 72°C for 1 minute; 72°C for 1 minute). Amplified cDNA was then used for both 5′ gene expression library construction and TCR enrichment. For gene expression library construction, 50 ng of amplified cDNA was fragmented and end-repaired, double-sided size-selected with SPRIselect beads, PCR-amplified with sample indexing primers (98°C for 45 seconds; 14–16 cycles of 98°C for 20 seconds, 54°C for 30 seconds, 72°C for 20 seconds; 72°C for 1 minute), and double-sided size-selected with SPRIselect beads. For TCR library construction, TCR transcripts were enriched from 2 mL of amplified cDNA by PCR (primer sets 1 and 2: 98°C for 45 seconds; 10 cycles of 98°C for 20 seconds, 67°C for 30 seconds, 72°C for 1 minute; 72°C for 1 minute). After TCR enrichment, 50 ng of enriched PCR product was fragmented and end-repaired, size-selected with SPRIselect beads, PCR-amplified with sample-indexing primers (98°C for 45 seconds; 9 cycles of 98°C for 20 seconds, 54°C for 30 seconds, 72°C for 20 seconds; 72°C for 1 minute), and size-selected with SPRIselect beads. The scRNA-Seq and scTCR-Seq libraries were sequenced on an Illumina NovaSeq 6000 to sequencing depth of 500 million reads and 60 million reads per sample, respectively. The sequencing data are available in NCBI’s Gene Expression Omnibus (GEO GSE188620).

### Processing of scRNA-Seq and TCR-Seq data.

The scRNA-Seq reads were aligned to the GRCh38 reference genome and quantified using Cell Ranger count (10x Genomics, version 4.0.0). Filtered gene-barcode matrices that contained only barcodes with unique molecular identifier (UMI) counts that passed the threshold for cell detection were used for further analysis. The scTCR-Seq reads were aligned to the GRCh38 reference genome, and consensus TCR annotation was performed using Cell Ranger vdj (10x Genomics, version 4.0.0). To identify the TCR clonotypes that were enriched in postvaccinated samples, the frequencies of each TCR clonotype (*TRA* and *TRB* combination) in prevaccinated and postvaccinated samples in each patient were compared, and the clonotypes for which frequencies in the postvaccinated sample were higher than the prevaccinated sample with adjusted *P* value less than 0.15 (calculated by Benjamini-Hochberg procedure) were defined as “enriched clonotype.”

All additional analyses were performed using Seurat (version 4.0.0) (38). Cells with less than 200 or greater than 3500 genes detected or greater than 10% mitochondrial RNA content were excluded from the analysis. Seurat objects were generated from raw UMI counts in each sample and counts data were log-normalized independently. The TCR information was added to the corresponding Seurat object using the AddMetaData function. For clustering of all cell types in PBMCs, variable genes and anchors were called on using the FindVariableFeatures and FindIntegrationAnchors functions, respectively, resulting in generating the integrated Seurat object. Scaled *z* scores for each gene were calculated using the ScaleData function and user input into a principal component analysis (PCA) based on variable genes. Clusters were identified using shared nearest neighbor–based (SNN-based) clustering based on the first 10 principal components with k = 30 and resolution = 0.3, and the same principal components were used to generate the uniform manifold approximation and projection (UMAP). Clusters were then annotated based on the expression of known marker genes (39). The cells in 5 clusters that represent T cell and NKT cell clusters were extracted and reclustered using SNN-based clustering based on the first 10 principal components with k = 30 and resolution = 0.2.

### Bulk TCR-Seq.

For bulk TCR-Seq in TILs, genomic DNAs were extracted from frozen resected tumor specimens using AllPrep DNA/RNA Mini Kit (Qiagen) per the manufacturer’s instructions. TCR-β complementarity-determining region 3 (CDR3) regions were amplified and sequenced from 2.5–3 mg of genomic DNA utilizing the immunoSEQ Assay (Adaptive Biotechnologies). Sequences were collapsed and filtered to identify and quantitate the absolute abundance of each unique TCR-β CDR3. To evaluate whether the T cells with enriched clonotypes in peripheral blood migrated into the TME, we assessed TCR-β overlap between TILs determined by bulk TCR-Seq and PBMCs determined by scTCR-Seq and visualized a Venn diagram using VennDiagram package on R Studio.

### Coculture of CD8^+^ T cells with DCs.

Human CD14^+^ cells and T cells were isolated from cryopreserved PBMCs using CD14 MicroBeads (Miltenyi Biotec) and EasySep Human T Cell Isolation Kit (STEMCELL Technologies), respectively. Monocyte DCs were generated from human CD14^+^ cells using CellXVivo Human Monocyte-Derived DC Differentiation Kit (R&D Systems). Immature DCs were incubated with or without either GBM6-AD lysate (10 μg/mL) or recombinant human EphA2 and IL-13Rα2 (10 μg/mL, Sino Biological) for 8 hours. DCs and T cells were cocultured at the ratio of 1:4 in X-VIVO supplemented with 2% human AB serum and 5 ng/mL of human IL-7 (PeproTech) for 5 days. CD8^+^ T cells were isolated from the cocultured cells using the CD8^+^ T-Cell Isolation Kit (Miltenyi Biotec). Genomic DNA was extracted from CD8^+^ T cells using NucleoSpin Tissue (Takara Bio) for bulk TCR-Seq.

### Tumor bulk RNA-Seq library preparation.

Total RNA was extracted from frozen resected tumor specimens using AllPrep DNA RNA Mini Kit (Qiagen) following the manufacturer’s protocol. RNA integrity was evaluated using Agilent Bioanalyzer 2100, and the samples with RIN 7 or greater were used for the following analyses. The following library preparation and sequencing were performed by DNA Technologies and Expression Analysis Core at University of California Davis (UC Davis) Genome Center. Strand-specific and barcode-indexed RNA-Seq libraries were generated from 300 ng total RNA each after poly-A enrichment using the mRNA-Seq Hyper Kit (Kapa Biosystems) following the instructions of the manufacturer. The fragment size distribution of the libraries was verified via microcapillary gel electrophoresis on a Bioanalyzer 2100. The libraries were quantified by fluorometry on a Qubit fluorometer (Life Technologies) and pooled in equimolar ratios. The pool was quantified by quantitative PCR with a Library Quant Kit (Kapa Biosystems) and sequenced on an Illumina NovaSeq 6000 with paired-end 150 bp reads. The sequencing data are available in GEO (GSE188620).

### Tumor bulk RNA-Seq data analyses.

Quality check (QC) and adapter-trimming were performed on the generated FASTQ sequencing data using fastq (v. 0.21.0) with default settings (40). An average of 180.8 million pass-QC sequencing read was obtained per tumor sample. Pass-QC reads were then mapped to the human reference genome hg19 (GRCh37.p13) using STAR (v. 2.5.4b) with the guidance of transcriptome annotation Homo_sapiens.GRCh37.87.chr.gtf. The subsequent sorting and indexing were carried out using samtools (v. 1.10). Gene-level expression abundance was calculated using StringTie (v. 2.1.4) as count and transcript-per-million (TPM) values (41).

### TCGA data analysis.

RNA-Seq gene expression data were downloaded through the UCSC Xena Toil web portal (data set ID: TcgaTargetGtex_rsem_gene_tpm; version: 2016-09-03; https://xenabrowser.net/datapages/?dataset=TcgaTargetGtex_rsem_gene_tpm&host=https%3A%2F%2Ftoil.xenahubs.net&removeHub=https%3A%2F%2Fxena.treehouse.gi.ucsc.edu%3A443); ref. 42). From the whole data set, 656 cases were extracted as subject to analysis (151 cases from TCGA-GBM and 447 cases from TCGA-LGG). We classified the cases into IDH-WT glioma (IDHwt); IDH-mutant, 1p19q–non-codeleted astrocytoma (IDH-A); and IDH-mutant, 1p19q-codeleted oligodendroglioma (IDH-O), based on their previously defined molecular diagnoses (43). The values in the downloaded data were then converted back to TPM values for subsequent analyses.

### Immune cell deconvolution analysis.

For immune cell composition prediction deconvolution, the TPM-summarized gene expression data were uploaded to and analyzed by CIBERSORTx with “absolute” mode and with quantile normalization disabled (15) (https://cibersortx.stanford.edu/). The analysis estimated the score of each of the samples as to the 22 distinct immune cell compositions (“LM22”), which can be compared among samples as well as cell types but does not represent the cell fraction. The scores were compared among the 3 molecular categories with the Kruskal-Wallis test with Holm’s multiple testing corrections followed by Dunn’s post hoc test.

### Statistics.

The statistical differences in the concentration of chemokines/cytokines in serum (Luminex) and the proportion of each cluster among each sample (CyTOF and scRNA-Seq) were calculated using a paired Wilcoxon test for longitudinal analysis (among arm 1 samples); a nonpaired Wilcoxon test was used for the direct comparison between arm 1 and 2 samples. The differences of the several markers’ expression on every single cell in CyTOF data were analyzed by a nonpaired, 2-tailed *t* test. PFS (time from A1 date, which is the date of the first adjuvant vaccine, to disease progression per RANO criteria) was estimated using Kaplan-Meier survival curves, and statistical differences were analyzed by log-rank test. Statistical analysis and data visualization were performed with R version 4.0.2 or GraphPad Prism version 6.01, and *P* less than 0.05 was regarded as statistically significant.

### Study approval.

This study (ClinicalTrials.gov NCT02549833) was approved by the IRB at UCSF and was conducted according to the Declaration of Helsinki. All patients provided written informed consent.

## Author contributions

JWT and H. Okada conceptualized and designed the study. H. Ogino, TN, PBW, KO, and A. Saijo analyzed PBMC and tumor tissue samples. JWT, NAB, JLC, NAOB, SLHJ, PT, SMC, and MSB recruited patients and managed clinical care. A. Shai, CMW, and JJP processed the tumor specimens and performed the pathological analyses. DG, MRT, and JER coordinated the trial and summarized the clinical data. AMM supervised the statistical and analytical methods used. MRO and CLM developed the GBM6-AD vaccine. AMS provided poly-ICLC. H. Ogino, JWT, TN, DG, PBW, KO, AS, MRT, AS, CMW, JER, MRO, CLM, YN, AMS, AMM, JJP, NAB, JLC, NAOB, SLHJ, PT, SMC, MSB, and H. Okada participated in writing and revising the manuscript.

## Supplementary Material

Supplemental data

Trial reporting checklists

ICMJE disclosure forms

## Figures and Tables

**Table 2:**
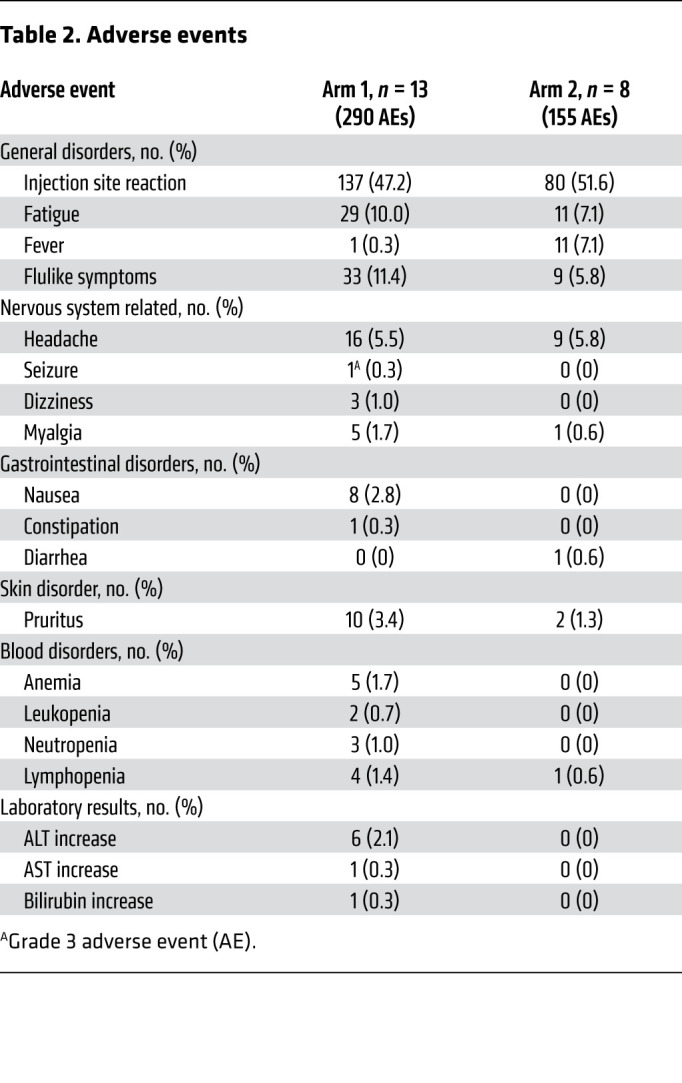
Adverse events

**Table 1:**
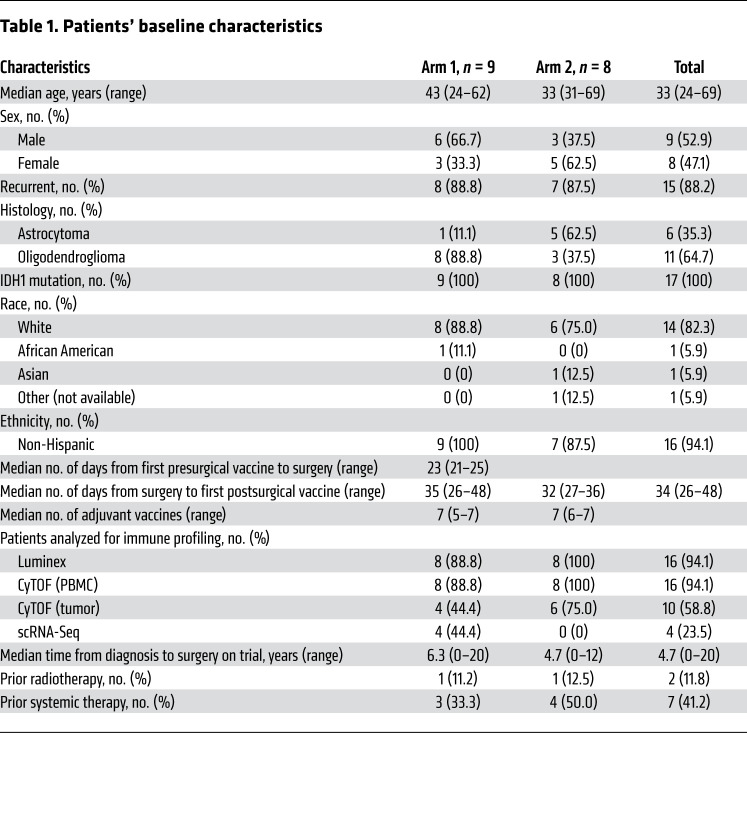
Patients’ baseline characteristics

## Version 1 12/09/2021

In-Press Preview

## Version 2 02/01/2022

Electronic publication
