# Supplementary material for: Randomized trial of neoadjuvant vaccination with tumor-cell lysate induces T cell response in low-grade gliomas
Source: J Clin Invest. 2022 Feb 1;132(3):e151239. doi: 10.1172/JCI151239 (PMC8803342; doi:10.1172/JCI151239)
Supplement: Supplemental data [file jci-132-151239-s103.pdf]

Supplemental Figure 1.

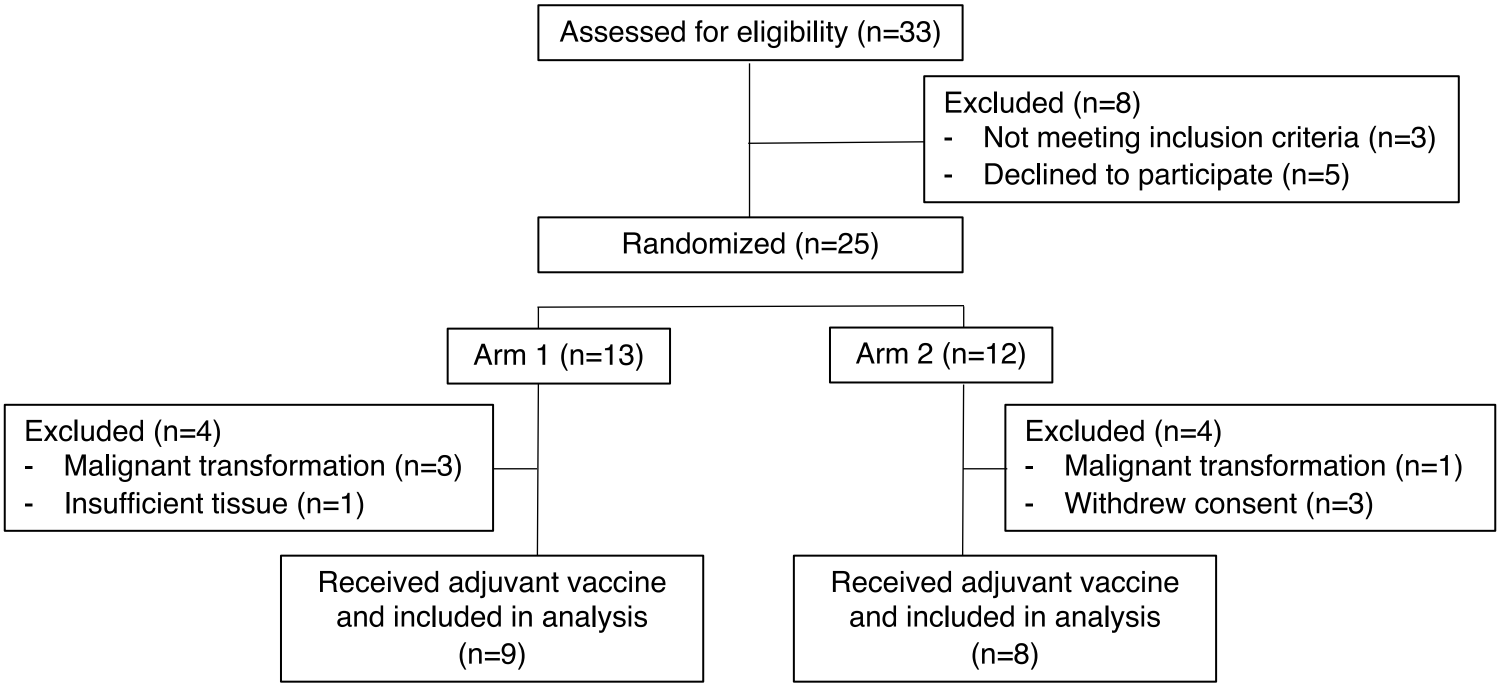

Supplemental Figure 1. The flow diagram of this study.

Supplemental Figure 2.

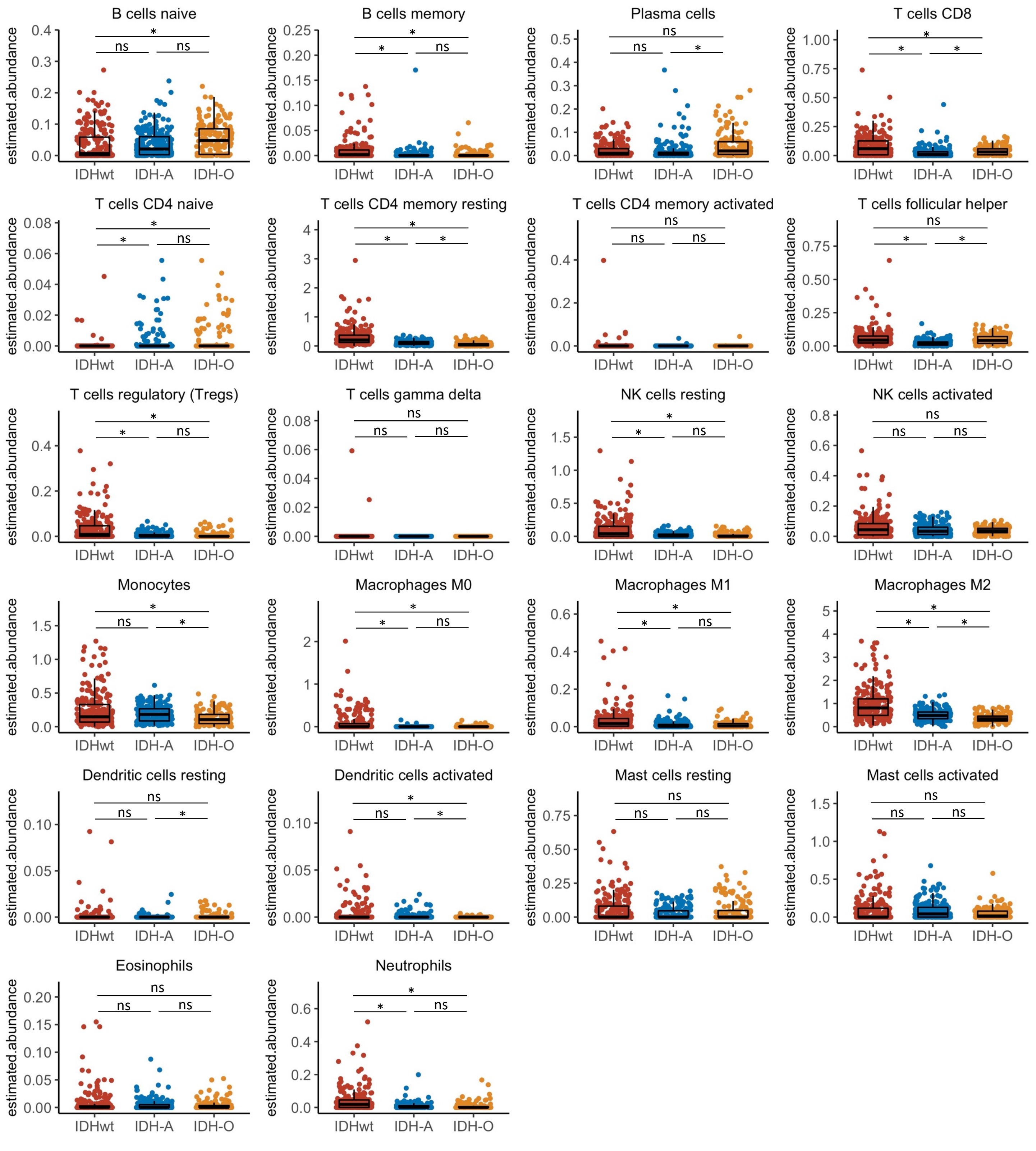

Supplemental Figure 2. Astrocytoma and oligodendroglioma have similar immune cell compositions in their tumor microenvironment. Bulk RNA-seq gene expression data of IDH-wildtype glioma (IDHwt), IDH mutant, 1p19q-non-codeleted astrocytoma (IDH-A), and IDH-mutant, 1p19q-codeleted oligodendroglioma (IDH-O) from TCGA were downloaded through the UCSC Xena Toil web portal, and analyzed by CIBERSORTx deconvolution. Although slight differences were observed for the densities of some immune cells between IDH-A and IDH-O, the differences were much more remarkable between IDHwt and IDH-mutant glioma. ns; non-significant, *p<0.05 (calculated by Kruskal-Wallis test with Holm's multiple testing corrections followed by the Dunn post hoc test).

Supplemental Figure 3.

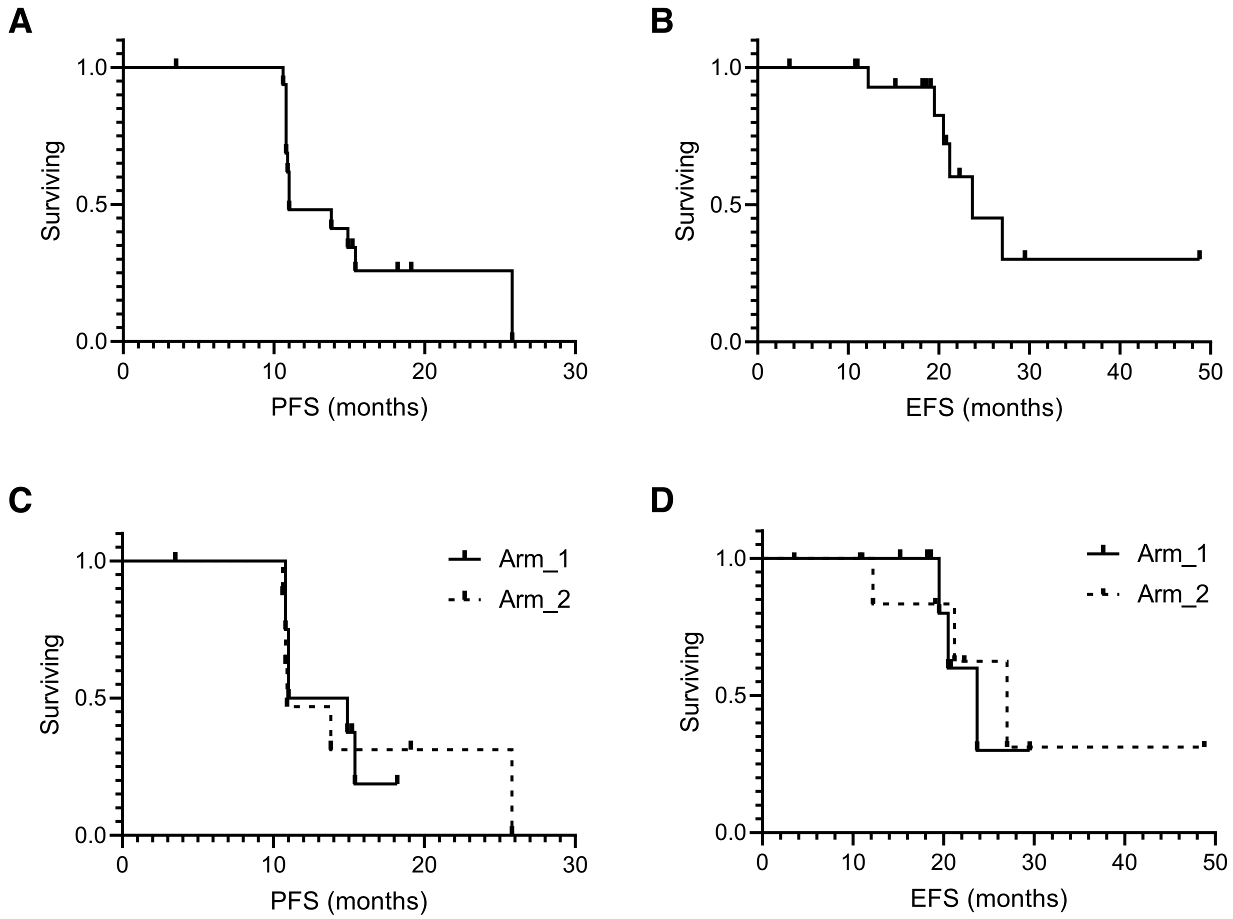

Supplemental Figure 3. Clinical outcomes of the patients in this study. (A) Progression free survival (PFS) was calculated from time of post-operative A1 vaccine to time of centrally confirmed imaging progression. Median PFS was 11.0 months (95% CI 10.8 – 15.4 months). **(B)** Event free survival (EFS) was calculated from time of post-operative A1 vaccine to time of new therapy. Median EFS was 23.7 months (95% CI 19.5 months – not reached). **(C, D)** There were no significant differences between PFS or EFS between Arms.

Supplemental Figure 4.

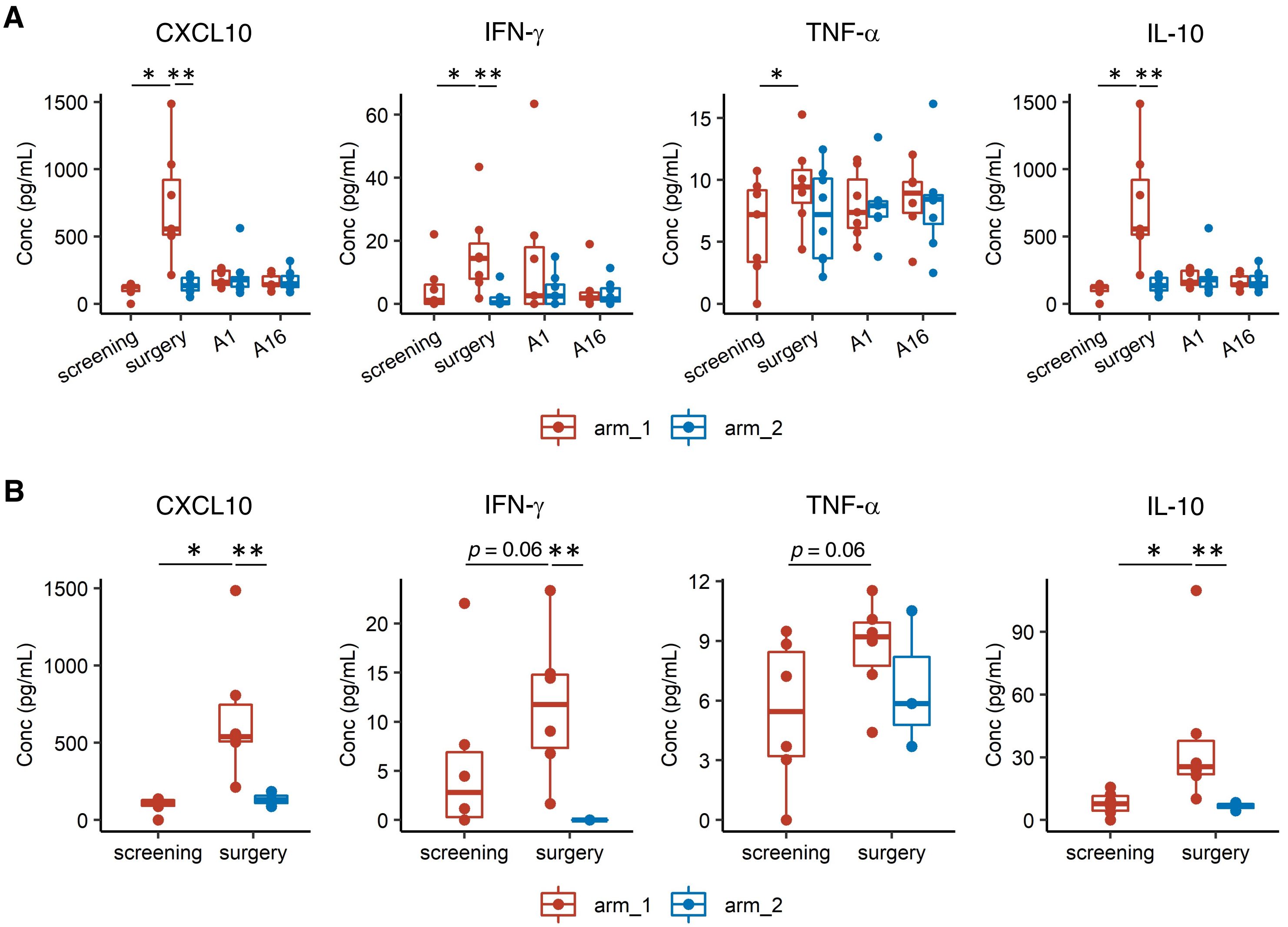

Supplemental Figure 4 (related to the data set in Figure 2). Upregulation of type-1 chemokines and cytokines induced by neoadjuvant vaccination was observed in Arm 1. (A) Serum concentrations of multiple chemokines and cytokines were measured by Luminex multiplex assay. The concentrations of type-1 chemokines and cytokines, such as CXCL10, IFN-γ, TNF-α, and IL-10, were elevated in Arm 1 samples on the day of surgery. On the other hand, none of them were upregulated in both arms on A16 date which are at three weeks after the most recent prior vaccination. **(B)** The same trends were observed when oligodendroglioma patients were selectively analyzed, though statistically significant differences in IFN-γ and TNF-α between the baseline and day of surgery in Arm 1 were lost because of the smaller number of cases. *$p<0.05$ (calculated by paired Wilcoxon test) and **$p<0.05$ (calculated by non-paired Wilcoxon test).

Supplemental Figure 5.

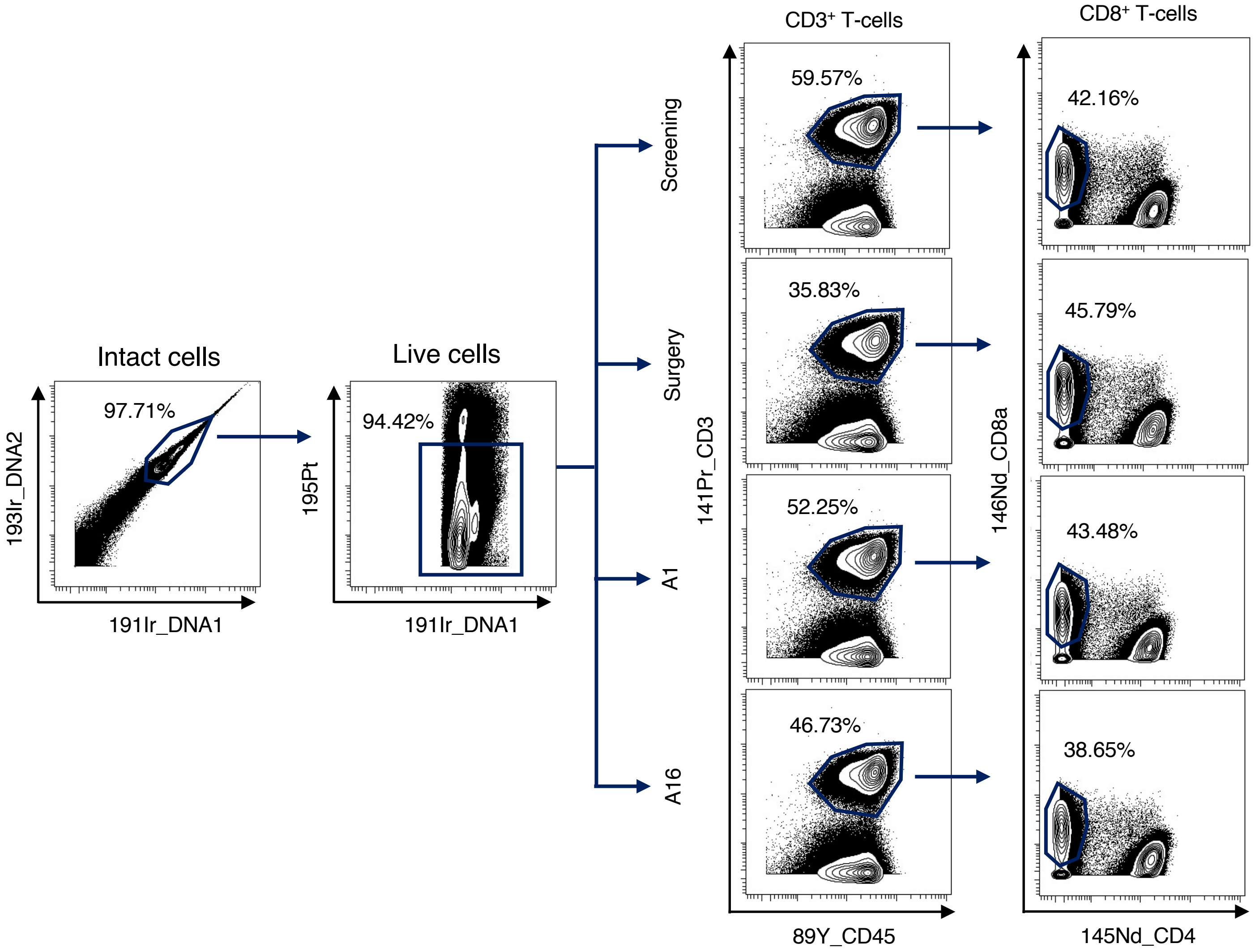

Supplemental Figure 5 (related to the data set in Figure 3). Gating strategy for CD8+ T-cells from PBMC on mass cytometric analyses. A representative gating strategy to identify CD8+ T-cells from PBMCs derived from one patient. Cells staining double-positive with iridium (Ir) intercalator in channels 191 and 193 were identified as intact cells (left). Cells with low cisplatin staining were identified as live cells (second to left). Cells were then debarcoded based on their three-metal combinations from barcoding reagents, and divided into samples from each time point. Cells staining positive for CD45 and CD3 were selected as T-cells (second to right), and positive for CD8a were identified as CD8+ T-cells (right).

Supplemental Figure 6.

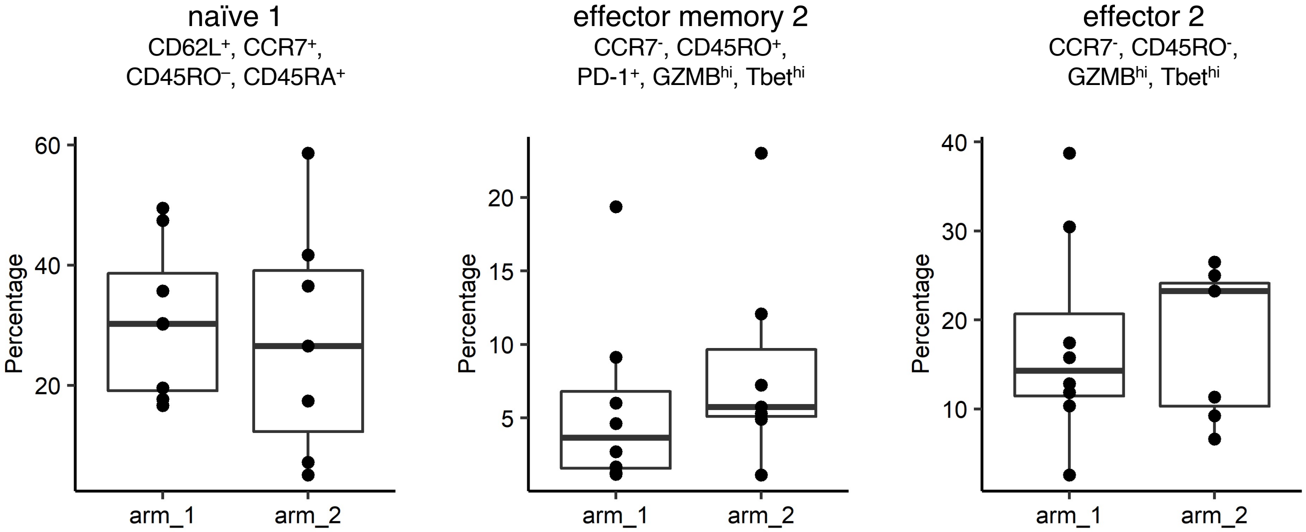

Supplemental Figure 6 (related to the data set in Figure 3). No differences in the cluster proportions were observed between Arm 1 and Arm 2 peripheral CD8+ T-cell samples on the day of surgery. In the mass cytometric analyses using peripheral CD8+ T-cells, the proportions of the clusters, which were significantly downregulated (naïve) or upregulated (PD-1+ GZMB^{hi} Tbet^{hi} effector memory and GZMB^{hi} Tbet^{hi} effector) by neoadjuvant vaccination in Arm 1 samples, were found insignificantly different between Arms 1 and 2 samples on the day of surgery.

Supplemental Figure 7.

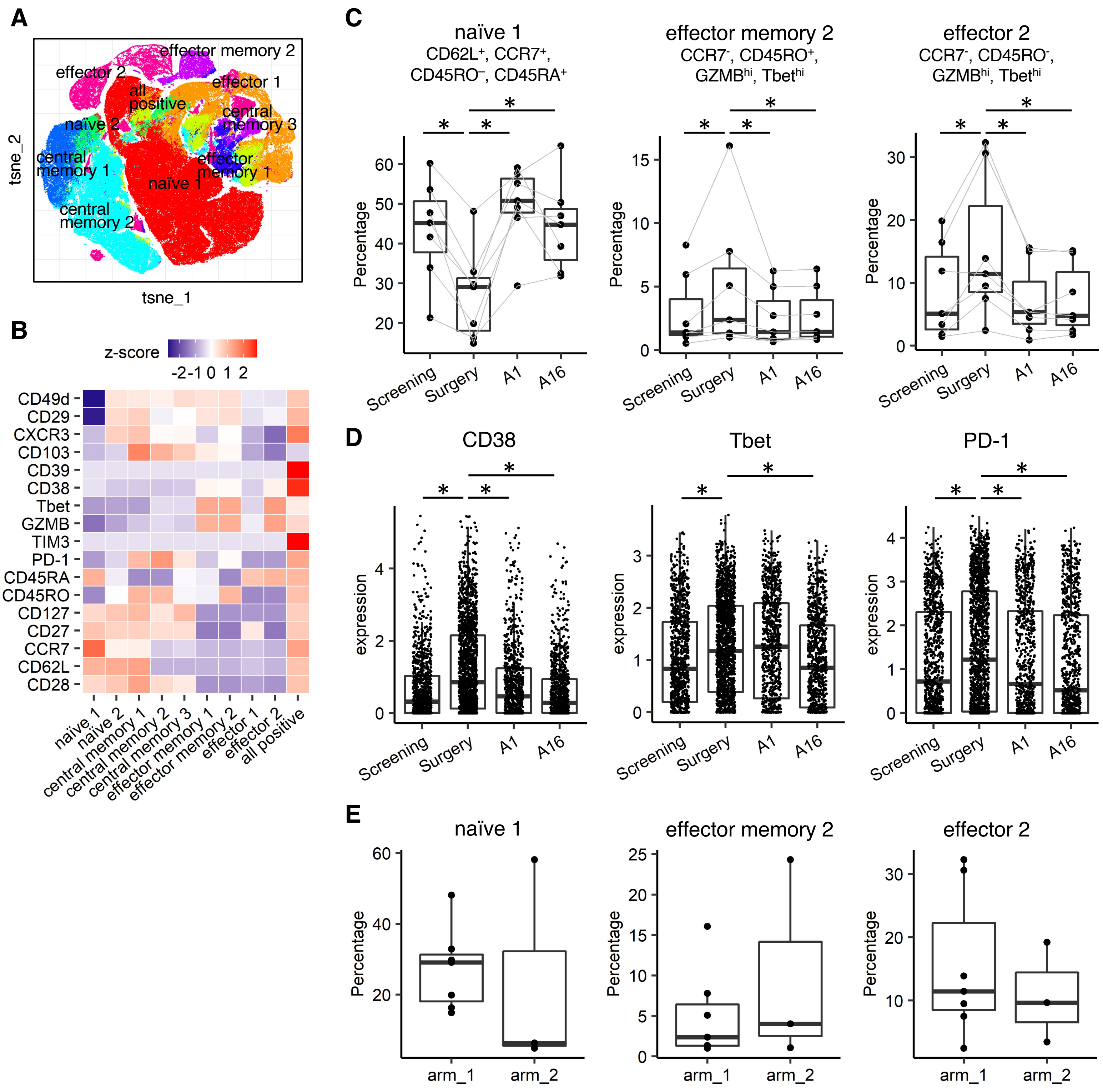

Supplemental Figure 7 (related to the data set in Figure 3). The same trends were observed in the dataset from only oligodendroglioma patients in mass cytometric analyses using PBMC. The mass cytometry dataset from oligodendroglioma patients (seven in Arm 1 and three in Arm 2) were selectively analyzed to evaluate the influence of the bias of tumor histology. **(A)** CD8+ T-cells were subjected to t-SNE for visualization and clustered by FlowSOM. **(B)** Heatmap visualizing the relative expression (Z score) of T-cell-relevant markers in each subpopulation. **(C)** Neoadjuvant vaccine with GBM6-AD and poly-ICLC increases GZMB^{hi} Tbet^{hi} effector memory and GZMB^{hi} Tbet^{hi} effector CD8+ T-cells while decreasing naïve CD8+ T-cells in Arm 1 patients. *$p<0.05$ (paired Wilcoxon test). **(D)** The expression of activation markers on every single cell in this GZMB^{hi} Tbet^{hi} effector memory cluster were enhanced in the post-neoadjuvant vaccinated samples. *$p<0.05$ (non-paired t-test). **(E)** No differences in the proportions of each cluster between Arm 1 and Arm 2 samples were seen on the day of surgery.

Supplemental Figure 8.

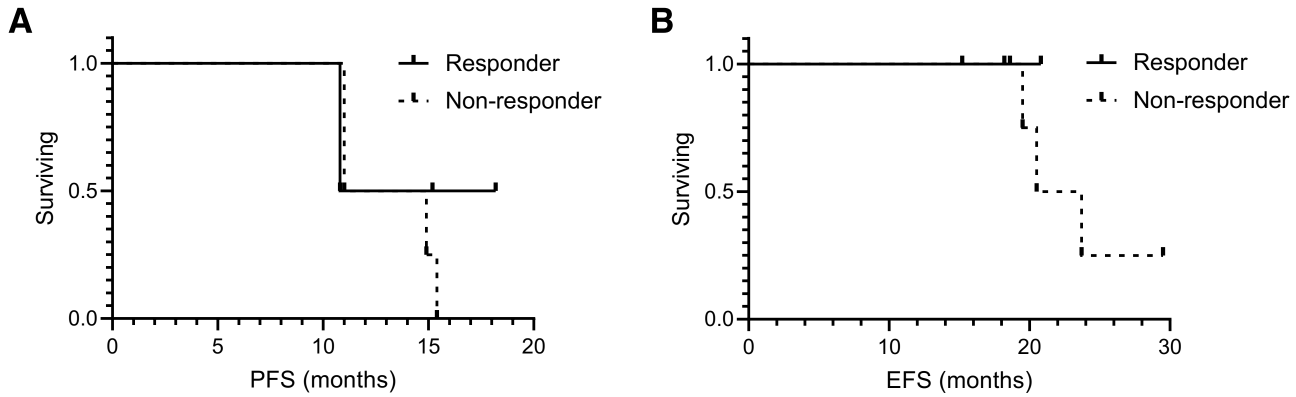

Supplemental Figure 8. The comparison of clinical outcomes between immunological responders and non-responders. Four patients who demonstrated a 10% or higher increase in the proportion of either PD-1^{+} GZMB^{hi} Tbet^{hi} effector memory or GZMB^{hi} Tbet^{hi} effector population following the neoadjuvant vaccination in peripheral blood, determined by CyTOF analyses, were defined as immunological responders (103-018, -026, -029, -051). There were not clear associations between the immunological response and (A) PFS or (B) EFS.

Supplemental Figure 9.

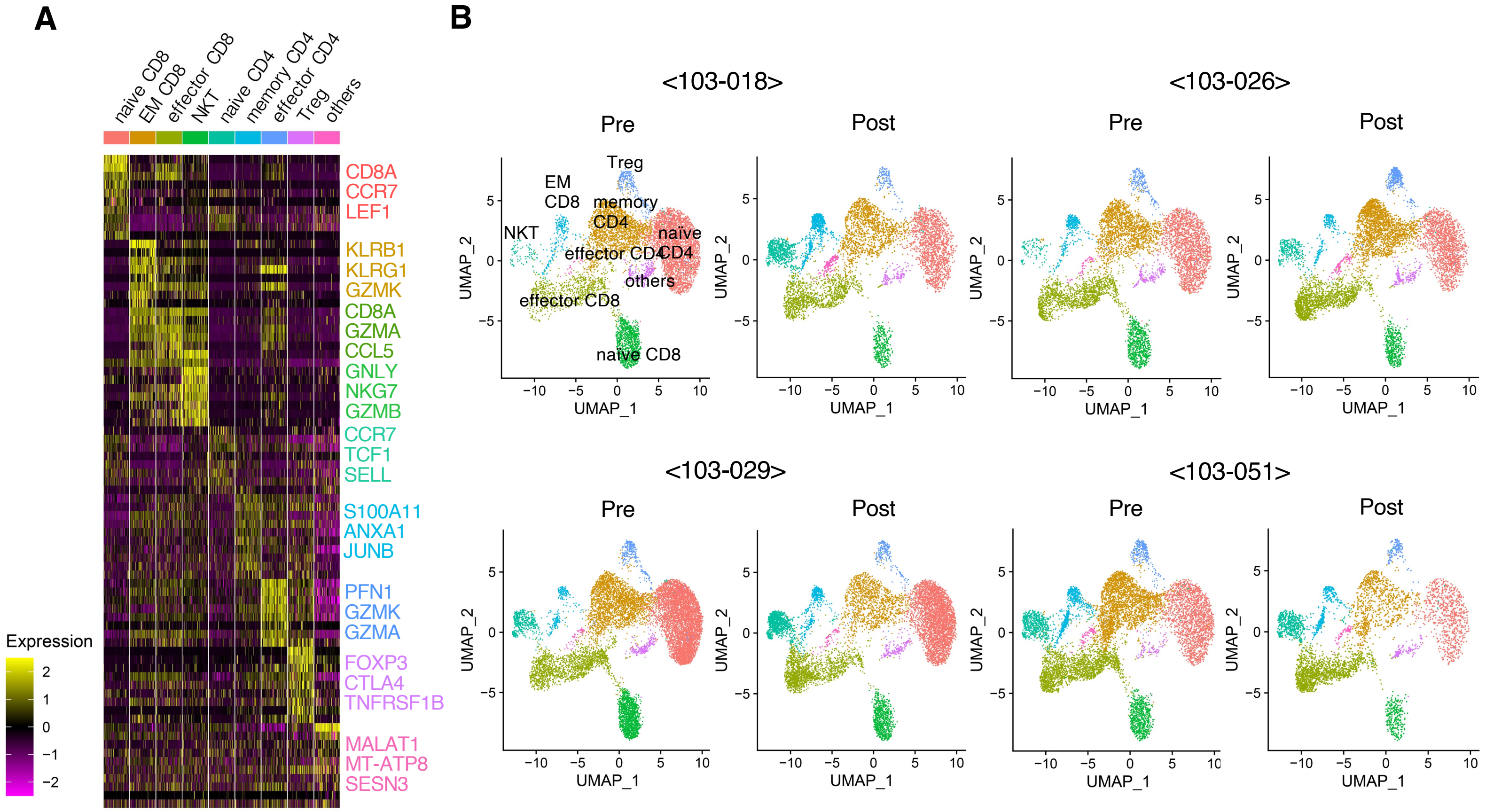

Supplemental Figure 9 (related to the data set in Figure 4). ScRNA-seq analyses showed the increases of effector CD4+ and CD8+, and decreases of naïve CD4+ and CD8+ T-cell populations in post-neoadjuvant samples. ScRNA-seq and scTCR-seq analyses were conducted using pre- and post-neoadjuvant vaccinated PBMCs from the four immunological responders, defined based on mass cytometric analysis (103-018, -26, -29, -51), on 10x GENOMICS platform. T- and NKT-cell populations were extracted from the original dataset, and re-clustered and grouped into nine subpopulations. **(A)** Heat map of differentially expressed genes between cells belonging to different T-cell subsets. Specific genes that are associated with different T-cell clusters are highlighted. **(B)** UMAP of T- and NKT-cells present in PBMC samples in each sample. The proportion of effector CD8+ T-cells and NKT-cells demonstrated a trend toward an increase in post-neoadjuvant vaccinated samples.

Supplemental Figure 10.

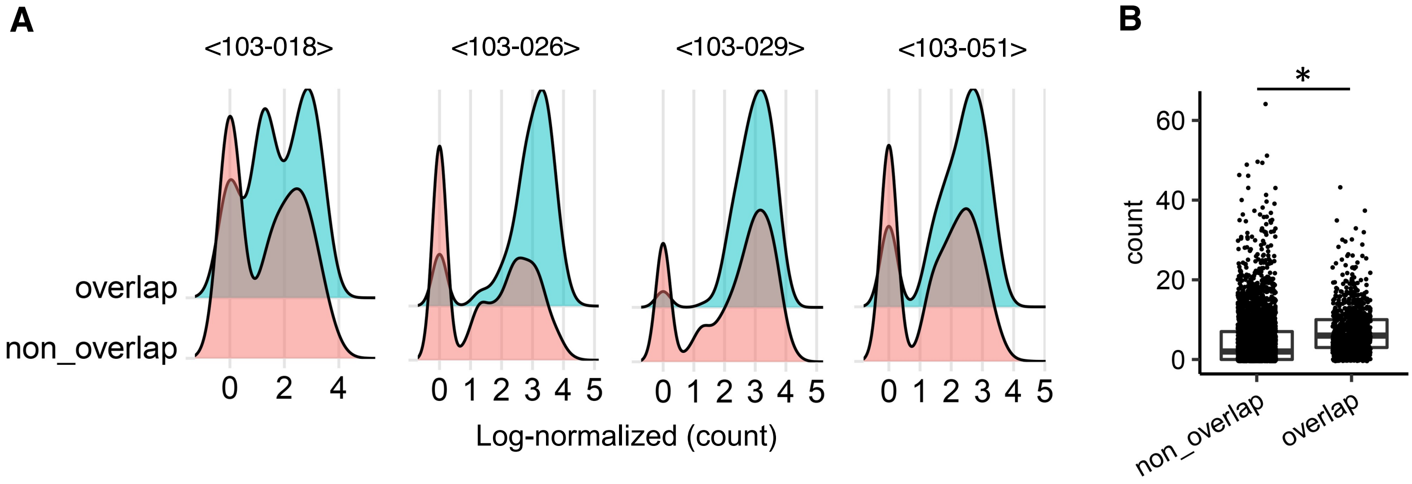

Supplemental Figure 10 (related to the data set in Figure 5). The upregulation of GZMB was more remarkable in the effector CD8+ T-cells that existed in both PBMC and the tumor than in the effector CD8+ T-cells that were enriched in PBMC but not found in the tumor. Within the effector CD8 cluster, the T-cells with overlapped clonotypes expressed a higher level of GZMB than other T-cells. Log-normalized (count) on x-axis was calculated as log (count / [total count of the cell] x 10,000 + 1). *p<0.05 (non-paired t-test).

Supplemental Figure 11.

A

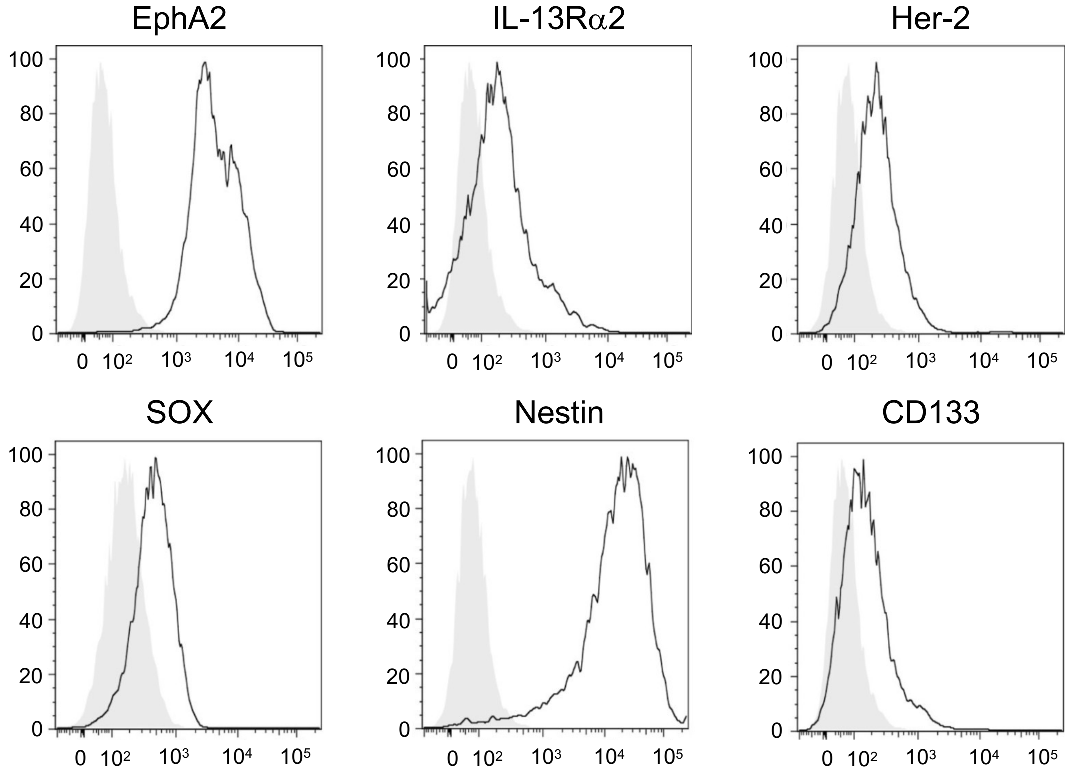

B

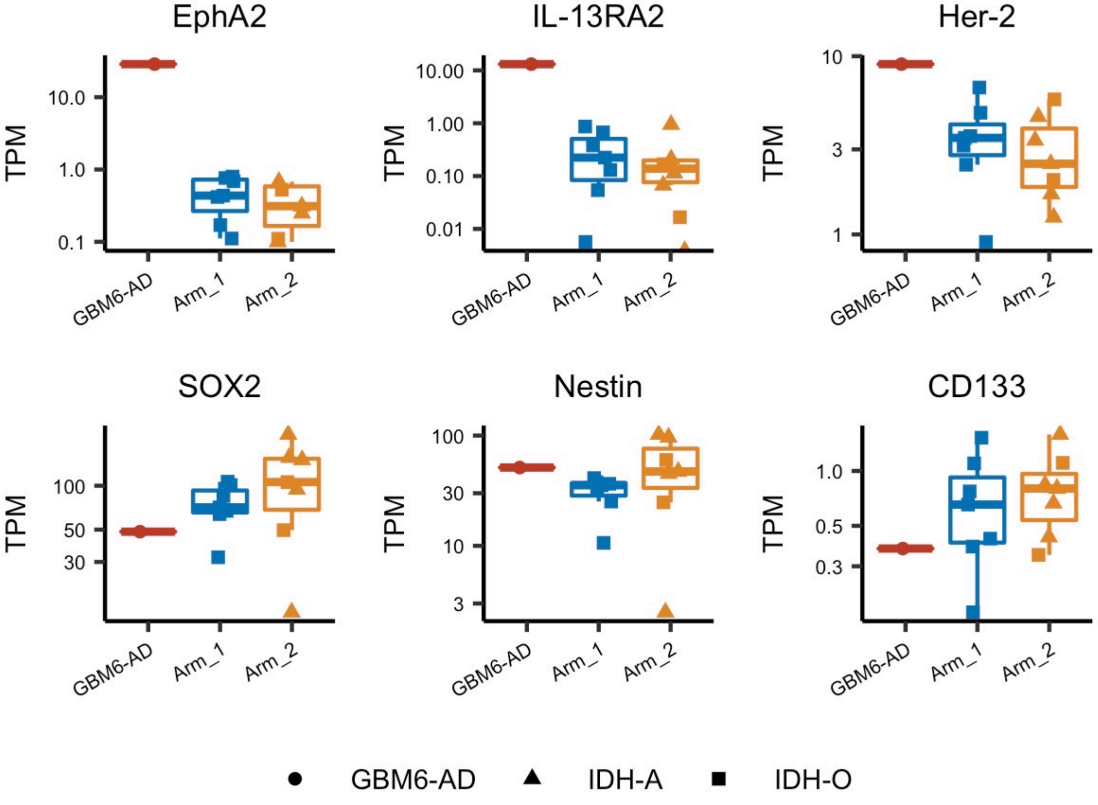

Supplemental Figure 11. The expression of glioma associate antigens (GAAs) or stem cell markers in GBM6-AD cells or tumor specimens. (A) Flow cytometry analyses demonstrated that GBM6-AD cells express not only several GAAs, such as EphA2, IL-13Rα2, and Her-2, but also several brain tumor initiating cells (BITCs) markers, such as SOX, Nestin and CD133. (B) Bulk RNA-seq analysis also demonstrated that both GBM6-AD and IDH-mutant LGG tumors express these antigens, albeit at different levels.

Supplemental Figure 12.

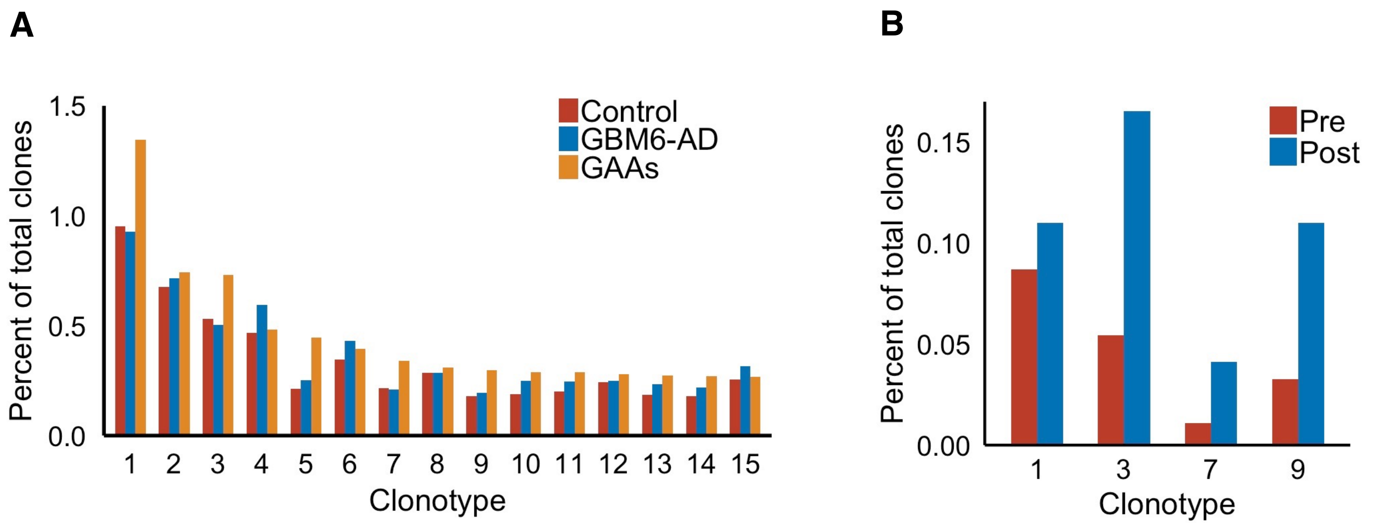

Supplemental Figure 12. Some of the TCR clones responded to the GBM6-AD lysate-derived antigens. (A) In patient 103-018, four of the top 15 most abundant $CD8^+$ T-cell-derived TCR clonotypes (i.e. clones 1, 3, 7, and 9) enriched more than 20% in response to GAA-stimulation when compared with the control group with no stimulation. However, a robust response of these TCR clones to GBM6-AD was not observed. **(B)** Nevertheless, scTCR-seq analyses showed that the frequencies of these four $CD8^+$ T-cell clones among total T-cells in peripheral blood increased following the vaccination of GBM6-AD in this patient.

Supplemental Figure 13.

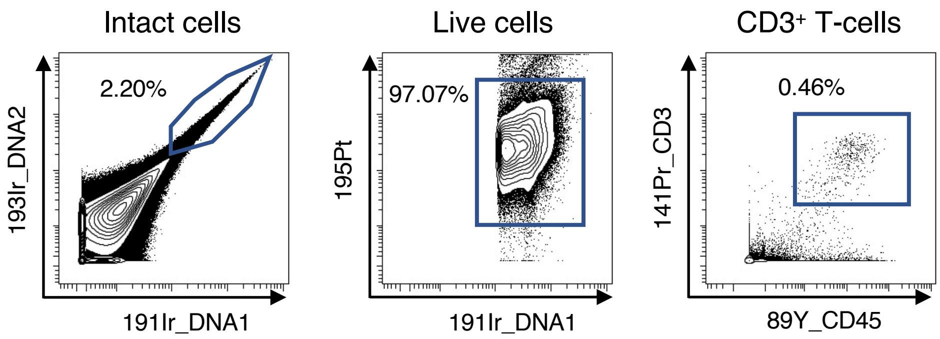

Supplemental Figure 13 (related to the data set in Figure 6). Gating strategy for mass cytometric analyses of CD3⁺ T-cells dissociated from tumor samples. A representative gating strategy to identify CD3⁺ T-cells from single-cell suspensions obtained from the tumor of one patient. These samples were not barcoded and stained independently. Cells staining double-positive with iridium (Ir) intercalator in channels 191 and 193 were identified as intact cells (left). Cells with low cisplatin staining were identified as live cells (middle). Cells staining positive for CD45 and CD3 were selected as CD3⁺ T-cells (right).

Supplemental Figure 14.

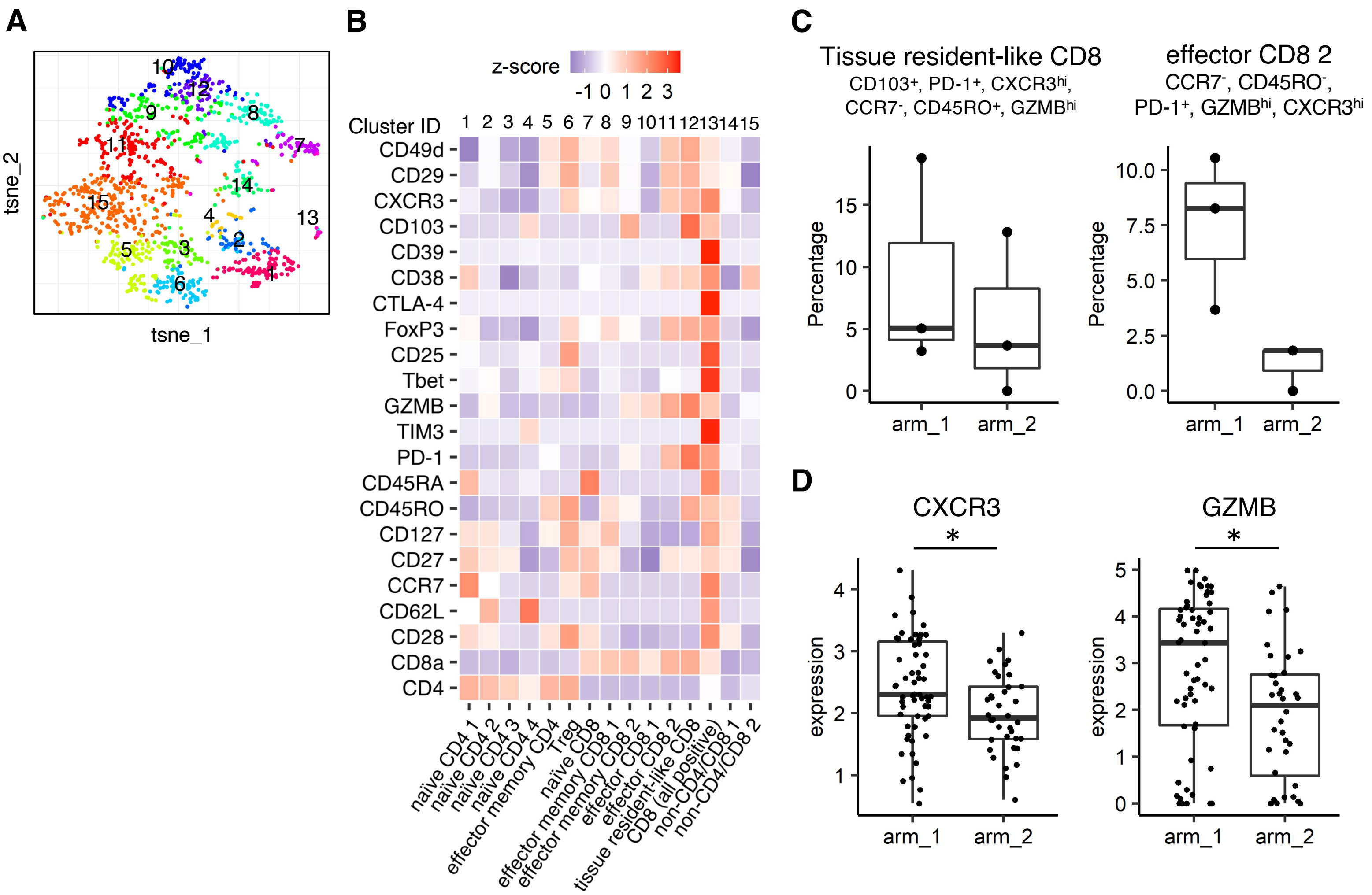

Supplemental Figure 14 (related to the data set in Figure 6). The same trends were observed in the mass cytometric analyses of tumor-infiltrating leukocytes when selected for oligodendroglioma patients only. The mass cytometry dataset from oligodendroglioma patients (three patients in each arm) was selectively analyzed for the potential bias of tumor histology. **(A)** $CD3^+$ T-cells were subjected to t-SNE and clustered by FlowSOM. **(B)** Heatmap visualizing the relative expression (Z score) of T-cell-relevant markers in each subpopulation. **(C)** The proportion of tissue resident-like $CD8^+$ T-cells with effector memory phenotype ($CD103^+$, $PD\text{-}1^+$, $CXCR3^{hi}$, $CCR7^-$, $CD45RO^+$, $GZMB^{hi}$) and effector $CD8^+$ T-cells showed a trend toward an increase in Arm 1 samples but without statistical significance. **(D)** TILs in this of tissue resident-like $CD8^+$ T-cells cluster in Arm 1 tumors demonstrated significantly higher expression levels for the CXCR3 and GZMB than those in Arm 2 tumors.

Supplemental Figure 15.

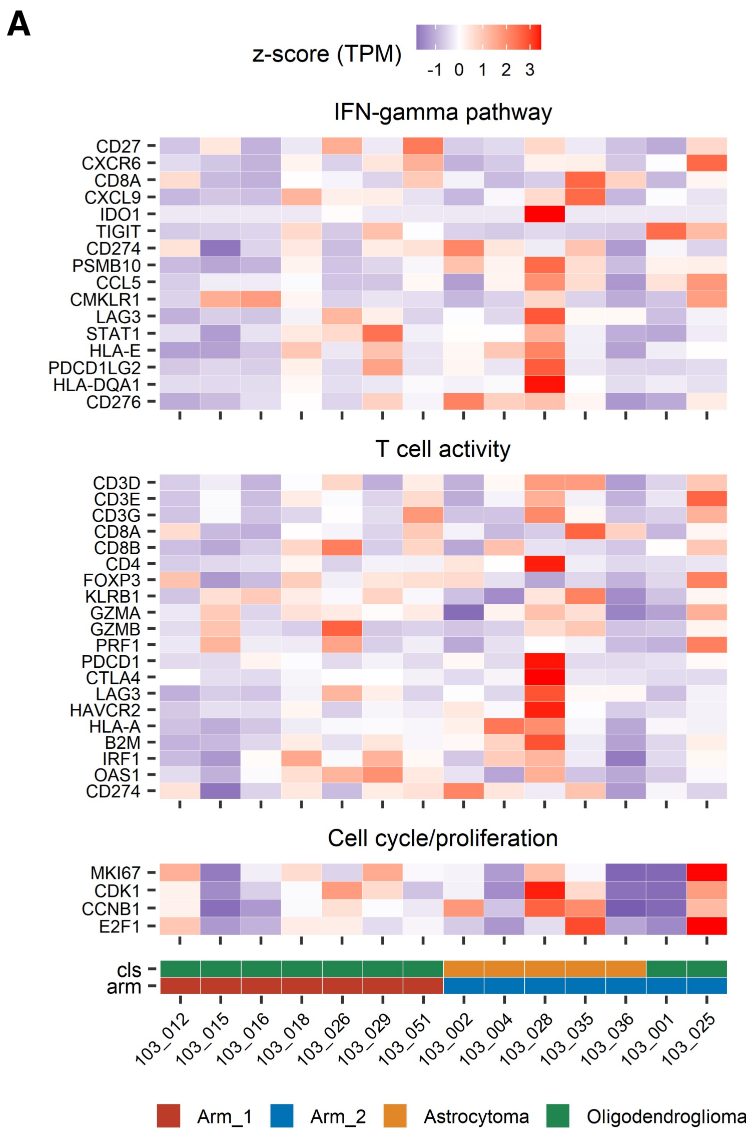

Supplemental Figure 15. The differences in the expression of immune-related genes between Arm 1 and Arm 2 tumors were not detected in bulk RNA-seq analyses. Bulk RNA-seq analysis using surgically resected tumor specimens was performed to evaluate the immune profile of TIL. However, the heat map, based on the relative TPM values of each immune related signature genes, didn’t show any differences in the expression patterns of these genes between Arm 1 and Arm 2 tumors.

Supplemental Table 1. Antibodies panel for mass cytometric analysis.

Label	Target	Clone	Vender	Cat #
89Y	CD45	HI30	Fludigm	3089003B
141Pr	CD3	UCHT1	Fludigm	3141019B
142Nd	CD19	HIB19	Fludigm	3142001B
143Nd	CD45RA	HI100	Fludigm	3143006B
144Nd	CD11b	ICRF44	Fludigm	3144001B
145Nd	CD4	RPA-T4	Fludigm	3145001B
146Nd	CD8	RPA-T8	Fludigm	3146001B
147Sm	CD11c	Bu15	Fludigm	3147008B
148Nd	CD14	RMO52	Fludigm	3148010B
149Sm	CD127	A019D5	Fludigm	3149011B
150Nd	CD86	IT2.2	Fludigm	3150020B
151Eu	CD103	Ber-ACT18	Fludigm	3151011B
152Sm	HLA-DR	L243	BioLegend	307651
153Eu	CD62L	DREG-56	Fludigm	3153004B
154Sm	TIM3	F38-2E2	Fludigm	3154010B
155Gd	PD-1	EH12.2H7	Fludigm	3155009B
156Gd	CD29	TS2/16	Fludigm	3156007B
157Gd	CXCR3	G025H7	BioLegend	353733
158Gd	CD27	L128	Fludigm	3158010B
159Tb	CX3CR1	2A9-1	BioLegend	341602
160Gd	CD28	CD28.2	Fludigm	3160003B
161Dy	Tbet	4B10	Fludigm	3161014B
162Dy	FoxP3	PCH101	Fludigm	3162011A
		259D/C7	Fludigm	3162024A
163Dy	CD33	WM53	Fludigm	3163023B
164Dy	CD45RO	UCHL1	Fludigm	3164007B
165Ho	CD163	GHI/61	Fludigm	3165017B
166Er	CD39	A1	BioLegend	328221
167Er	CCR7	G043H7	Fludigm	3167009A
168Er	CD206	15-2	Fludigm	3168008B
169Tm	CD25	2A3	Fludigm	3169003B
170Er	CTLA-4	14D3	Fludigm	3170005B
171Yb	CD68	Y1/82A	Fludigm	3171011B
172Yb	CD38	HIT2	Fludigm	3172007B

173Yb	GZMB	GB11	Fludigm	3173006B
174Yb	CD49d	9F10	Fludigm	3174018B
175Lu	PD-L1	29E.2A3	Fludigm	3175017B
176Yb	CD56	NCAM16.2	Fludigm	3176008B
209Bi	CD16	3G8	Fludigm	3209002B
